# Tackling Orientational Isomerism in Metal–Organic Frameworks Comprising Low‐Symmetry Linker Molecules via Simulated Annealing Featuring a Neural Network Potential

**DOI:** 10.1002/jcc.70349

**Published:** 2026-04-02

**Authors:** Benedikt E. Hörfarter, Stefan Seiwald, Clemens Hofstötter, Armin Penz, Josef M. Gallmetzer, Thomas S. Hofer

**Affiliations:** ^1^ Institute of General, Inorganic and Theoretical Chemistry University of Innsbruck Innsbruck Austria

**Keywords:** linker‐related orientational isomerism, metal–organic frameworks, Monte Carlo, neural network potential, simulated annealing

## Abstract

The perplexing structural diversity of metal–organic frameworks (MOFs) leads to astonishing unique properties and versatile potential technological applications of this porous materials class. However, whenever the organic linker molecules in a specific MOF compound may adopt multiple spatial orientations in‐between the inorganic nodes they coordinate to, a near infinite number of possible MOF configurations arises through orientational isomerism. The latter is particularly challenging to account for in computational studies. In this work, we present a novel simulated annealing (SA) approach via Monte Carlo (MC) runs to gain insights into linker molecule orientations within such compounds, at the example of the three increasingly complex MOF systems MOF‐5‐OH, SNU‐70 and UiO‐66(Zr)‐NH_2_. We thereby successfully identify reasonable approximations to the global minimum structures for all MOFs with regard to collective linker molecule orientations by performing random re‐orientation attempts of a randomly chosen organic linker in each MC step. The efficiency of the simulated annealing procedure is improved and guaranteed by exploiting the accuracy and speed of the state‐of‐the‐art neural network potential (NNP) MACE‐MP‐0a in all energy and force calculations. Critically, a substantial potential energy gain is observed during annealing, accompanied by systematic structural changes within the MOF compounds. The combined results of these energetic and structural characteristics highlight how it is indeed cooperative effects arising from the organic linker molecules that play a decisive role in determining the most favorable MOF configuration. Intriguingly, root‐mean‐square‐deviation (RMSD) calculations based exclusively on atoms of the inorganic nodes are found to be sensitive structural descriptors for tracking linker molecule re‐orientations, while powder X‐ray diffraction (PXRD) patterns and radial distribution functions (RDFs) derived from molecular dynamics (MD) simulations at standard conditions show less distinctive trends. In summary, the utility of the presented simulated annealing strategy is justifiable by its broad applicability for investigating linker‐related orientational isomerism in any relevant MOF as well as the possibility for studying even more complex host–guest systems in a similar manner.

## Graphical abstract

A robust and adaptable strategy for addressing orientational isomerism in metal–organic framework compounds with low‐symmetry linkers is presented, utilizing a combined neural network potential and simulated annealing approach.

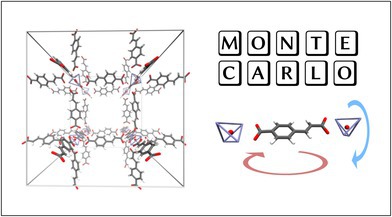

## 1 Introduction

Metal–organic framework (MOF) compounds represent a comparably young class of synthetic porous materials composed of inorganic coordination sites, typically metal ions or metal chalcogenide clusters, which are connected via organic linker molecules [1, 2]. MOFs feature a vast range of structural topologies and tunable properties, making them promising candidates for a wide array of applications including gas storage and separation [3], catalysis [4], sensing and detection [5], energy storage [6], drug delivery [7], as well as water purification [8]. The significance of this materials class is underscored by the 2025 Nobel Prize in Chemistry [9], awarded jointly to Susumu Kitagawa, Richard Robson, and Omar M. Yaghi for foundational advances in reticular chemistry underlying metal–organic framework design. The possibility to combine any suitable metal node with compatible linker molecules results in a near‐infinite number of possible MOF structures. As a consequence, computational methods to investigate the properties of novel or even hypothetical MOF compounds received increased attention in recent years [10, 11, 12, 13].

**FIGURE 1:**
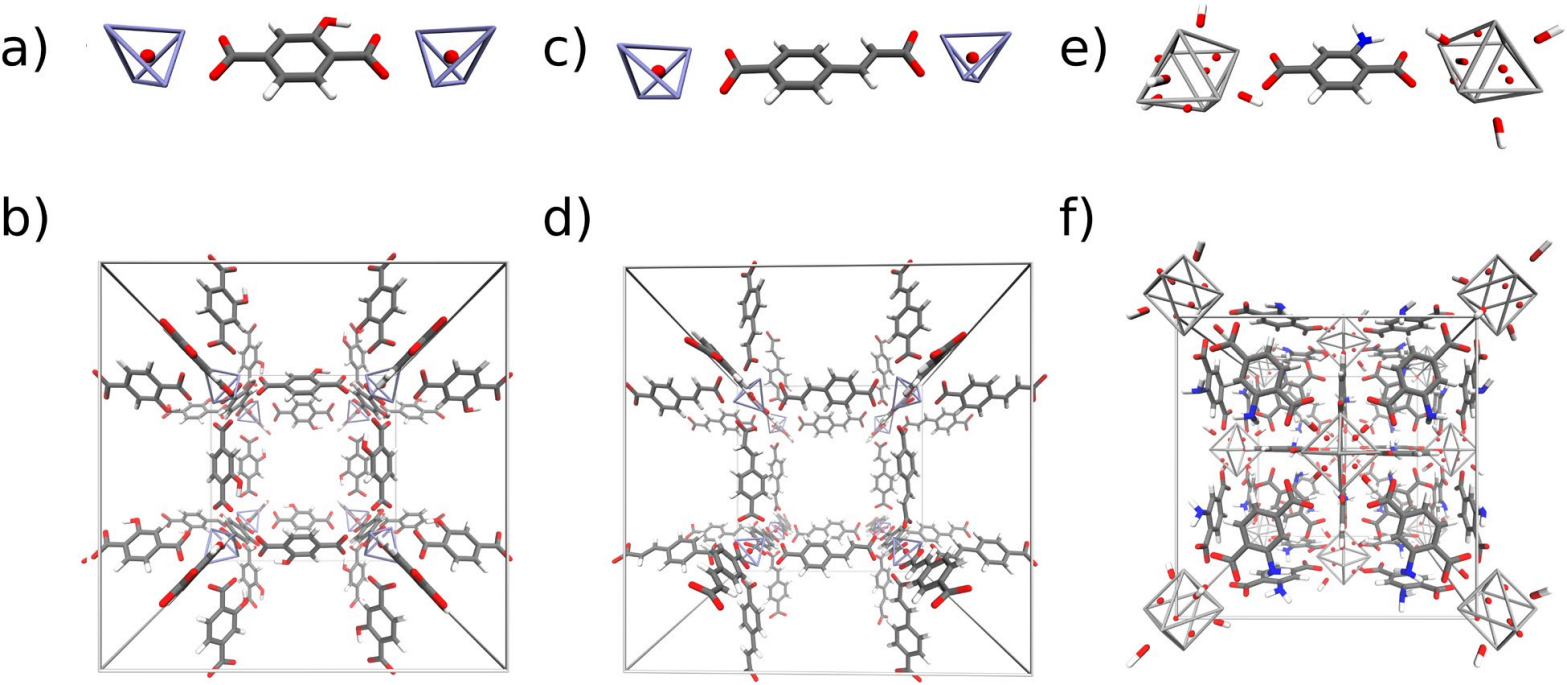
Illustrations of the investigated MOF systems comprising low‐symmetry linker molecules. (a) Structure of a BDC‐OH linker molecule coordinated to two Zn_4_O^6+^ inorganic nodes. (b) Initial crystal structure of the MOF‐5‐OH system. (c) Structure of a CVB linker molecule coordinated to two Zn_4_O^6+^ inorganic nodes. (d) Initial crystal structure of the SNU‐70 system. (e) Structure of a BDC‐NH_2_ linker molecule coordinated to two ${\text{Zr}}_{6}{\text{O}}_{4}{\text{(OH)}}_{4}^{12+}$ inorganic nodes. (f) Initial crystal structure of the UiO‐66(Zr)‐NH_2_ system. Atom colors: C—gray, H—white, O—red, N—blue, Zn—iceblue, Zr—metallic silver.

A particular challenge in the setup of computational studies is associated with the orientation of the linker molecules within MOF systems. While in case of symmetric linkers such as the widely used benzene‐1,4‐dicarboxylate (BDC) [14, 15, 16] a re‐orientation of the linker by rotation or mirroring has no impact on the structural composition of the MOF, introducing an asymmetry such as substituting one of the aromatic hydrogen atoms in BDC results in four possible orientations of the linker when coordinated to two inorganic nodes. This is demonstrated in case of (*E*)‐4‐(2‐carboxylatovinyl)benzoate (CVB), which is a building block of the MOF SNU‐70 [17] (see Figure 1). Due to the orientational anisotropy of the linker molecules in an experimental sample, it has not been possible to obtain an unambiguous crystal structure of this compound (see, for instance, CCDC‐846935) [17, 18]. Instead, a superposition of the four potential orientations has been determined in structure elucidation (see Supporting Information, Figure S1). A similar scenario concerning manifold possible configurations arises for instance in the MOF compounds MOF‐5‐OH [19] and UiO‐66(Zr)‐NH_2_ [20] (e.g., CCDC‐1507786) [18, 21] where instead of BDC its hydroxy‐ and amino‐substituted variants, i.e., 2‐hydroxybenzene‐1,4‐dicarboxylate (BDC‐OH) and 2‐aminobenzene‐1,4‐dicarboxylate (BDC‐NH_2_), are present (see Figure 1).

These undetermined structural properties are a particular challenge when generating initial structures in computational studies. One possible option is to assume a purely stochastic distribution by randomizing the orientations. However, this strategy immediately outrules any cooperative effects, such as an alternating variation in the orientation to compensate for potential misalignments in the interaction between linkers and inorganic nodes in the periodic system.

A straightforward brute‐force search for the energetically most favorable structure, that is a systematic variation considering all possible linker orientations, is unfeasible in most cases. For the linker species BDC‐OH, CVB, and BDC‐NH_2_ incorporated in MOF‐5‐OH, SNU‐70, and UiO‐66(Zr)‐NH_2_ a total of approximately 2.8 ⋅ 10^14^ possible linker permutations in each case. While this number can be reduced by symmetry operations by a factor of 2^3^ × 4^3^ = 512 due to mirroring and rotation along the principal directions, a systematic search considering all remaining variants remains impractical.

A much more efficient option is to employ random variations in the orientation of the linker molecules followed by a suitable acceptance criterion. Amirjalayer and Schmid have reported on a genetic algorithm to adress conformational isomerism within the isoreticular metal–organic framework (IRMOF) family [22]. Moreover, Monte Carlo (MC) methods based on the Metropolis criterion [23] are widely regarded as an effecitve tool to probe a wide conformational space in an efficient manner [24, 25]. Thus, in this work, a simulated annealing procedure [26, 27] using such an MC‐typical Metropolis algorithm to (globally) optimize the orientational distribution of low‐symmetry linker molecules in MOF structures is outlined and discussed at the example of the above mentioned MOF‐5‐OH, SNU‐70, and UiO‐66(Zr)‐NH_2_ MOF systems. To yield reliable results while keeping computation times manageable (i.e., quantum mechanical accuracy at force field cost), this simulated annealing procedure is carried out in conjunction with the state‐of‐the‐art neural network potential (NNP) MACE‐MP‐0a [28, 29]. The latter is based on the MACE (multi‐atomic cluster expansion) architecture [30, 31] and has shown excellent out‐of‐distribution performance for a wide variety of solid‐state systems including MOF compounds [28, 32, 33, 34].

## 2 Methods

A simulated annealing procedure [26, 27] based on the MC‐typical Metropolis algorithm [23], interfaced with the recently developed MACE‐type NNP MACE‐MP‐0a [28, 29] for energy and force calculations, was employed for global MOF geometry optimization with respect to orientational isomerism of low‐symmetry linker molecules, thereby applying periodic boundary conditions.

Upon reading in the initial MOF structure as well as after each attempted linker molecule rotation by 180° about a randomly chosen internal axis, as performed in each MC step attempt (*vide infra*), the system was relaxed by means of geometry optimization also considering the lattice parameters while at the same time keeping the angles within the unit cell fixed. The associated acceptance criterion for the rotation attempt was based on the Metropolis criterion [23]: In case the energy difference ΔE between the previously accepted step and the current step evaluated to ΔE<0 or the inequality exp(−ΔE/RT)>U was fulfilled, the step was accepted, otherwise it was rejected and restored. In the latter expression, R, T, and U denote, respectively, the ideal gas constant, temperature, and a uniformly distributed random number between 0 and 1, that is, U∼U(0,1).

**FIGURE 2:**
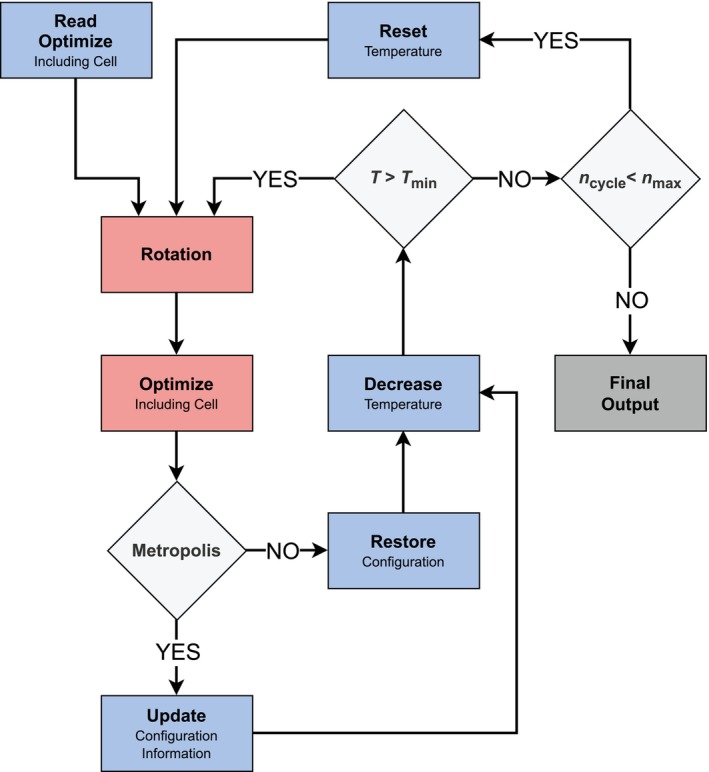
Flowchart of the NNP‐based simulated annealing algorithm to optimize the orientational distribution of low‐symmetry linker molecules in MOF systems. The algorithm consists of a random selection of a linker molecule and a subsequent rotation by 180° about either the plane, axial, or normal axis (randomly chosen), followed by geometry optimization including also the lattice parameters (typically keeping angles fixed). In an MC‐like fashion, the Metropolis criterion is applied to accept or reject the step attempt. The procedure is repeated until the temperature T has reached the final value $T_{\min}$ and the running index $n_{\text{cycle}}$ has reached the preset number of cycles $n_{\max}$.

Starting from a user‐defined initial value, the temperature T was decreased after each rotation attempt—regardless of acceptance or rejection—according to a cooling schedule until the final temperature $T_{\min}$ had been reached. Then, the temperature was reset to a defined starting value and the procedure was repeated, until a predefined number of cycles $n_{\max}$ (referred to as thermocycles) had been reached (with running index $n_{\text{cycle}}$). A flowchart summarizing the individual steps of the algorithm is depicted in Figure 2.

**FIGURE 3:**
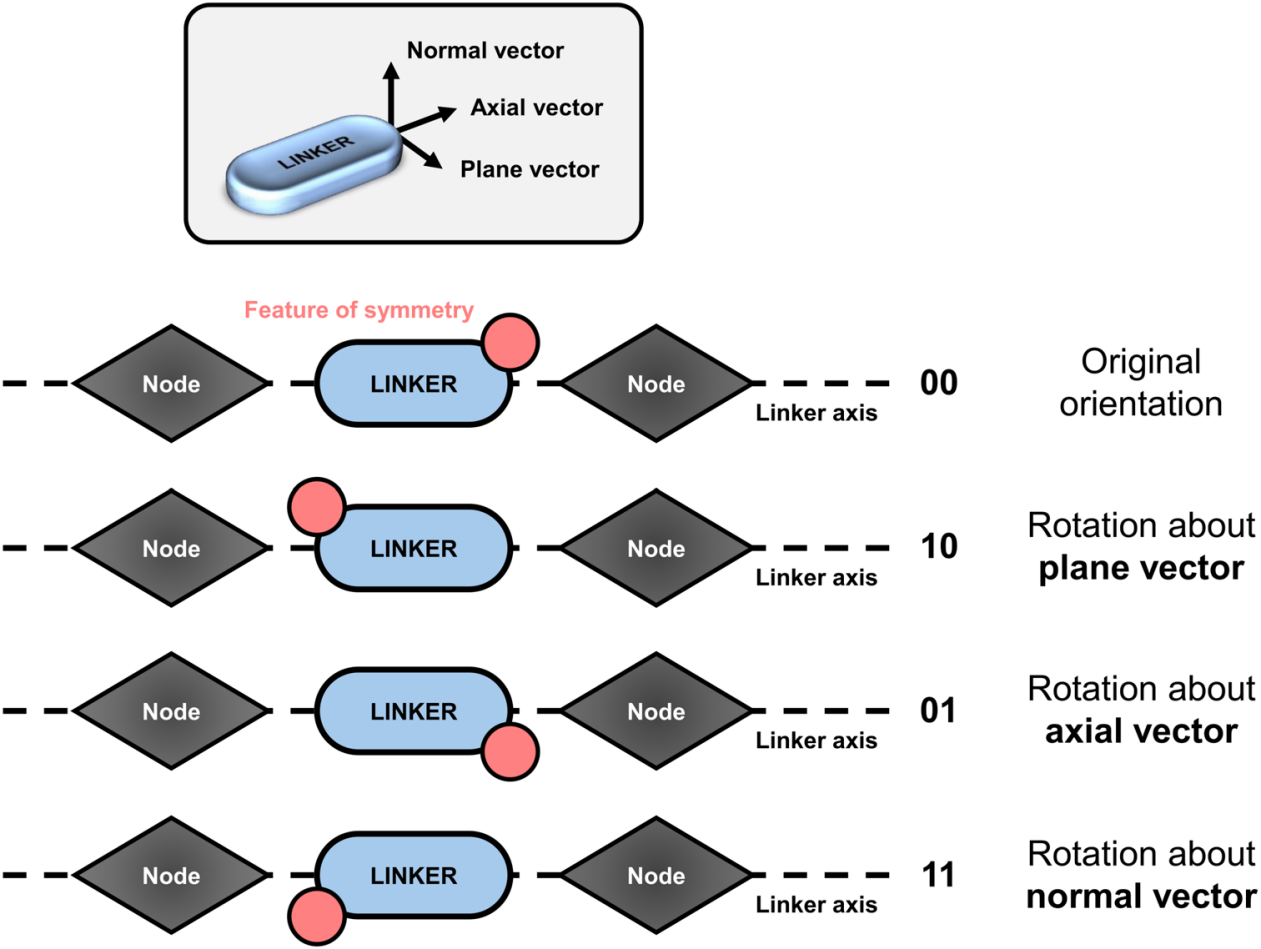
Depiction of the four possible orientations of an asymmetric linker molecule in‐between two MOF nodes (i.e., inorganic coordination sites). The possible rotations about either the plane, axial, or normal vector are shown including the corresponding 2‐bit binary representations for describing nodal and axial orientation.

The internal axes for linker rotations within each individual MC step attempt were calculated by diagonalizing the covariance matrix of the carbon atoms of the randomly selected linker molecule and defined via the thereby resulting so‐called plane, axial, and normal vectors, as displayed in Figure 3. Depending on the performed rotation, the linker molecule was re‐oriented in different ways, which was encoded in form of a 2‐bit binary representation, also shown in Figure 3. This 2‐bit representation allows tracking and distinguishing between flips where the feature of symmetry changes the node it coordinates to (change in nodal orientation via rotation about the plane vector) and flips that only affect the linker's spatial arrangement (change in axial orientation via rotation about the axial vector). In case of rotation about the normal vector, both the nodal and axial orientations are altered, reflected by switching both digits of the 2‐bit binary.

For analyzing the structural changes within the MOF systems during the simulated annealing procedure, root‐mean‐square‐deviation (RMSD) calculations [35] were performed considering only the atoms of the inorganic building units. Additionally, ensemble‐averaged powder X‐ray diffraction [36] (PXRD) patterns and radial distribution functions (RDFs) of various element pairs were calculated at ambient conditions for both the initial and optimized final structures of the MOF systems via separately performed molecular dynamics (MD) simulations. While the PXRD patterns were thought to provide insight into the long‐range order within the systems, the RDFs were expected to characterize changes in local structural features.

## 3 Computational Setup

Simulated annealing minimization was performed using the NNP MACE‐MP‐0a (model size medium) [28, 29] to compute energies and forces as implemented in the ASE Python package [37]. For exact reproducibility of the performed calculations, double‐precision floating‐point numbers were employed. To properly account for dispersion interactions, DFT‐D3 dispersion corrections with Becke–Johnson (BJ) damping [38] were added.

The initial structures of the MOF systems were constructed to represent collectively ordered orientations of the linker molecules for the MOF‐5‐OH, SNU‐70, and UiO‐66(Zr)‐NH_2_ MOF systems (see Figure 1), that is, without directional bias. Due to the ambiguity of such structures, the chosen input geometries shall be briefly highlighted. On the one hand, the SNU‐70 MOF system was constructed so that alternating inorganic nodes, Zn_4_O^6+^, were coordinated by six CVB molecules either pointing all towards or all away from the node with their alkene‐featuring side chains. The same pattern was realized in case of MOF‐5‐OH, with the hydroxy groups of the six BDC‐OH linker molecules jointly pointing towards or away from the individual Zn_4_O^6+^ nodes.

On the other hand, such an alternating pattern cannot be realized in the case of UiO‐66(Zr)‐NH_2_ due to its inherently different topology. Here, the BDC‐NH_2_ linker molecules were oriented so that in each direction (xy, xz, and yz planes), the amino groups collectively pointed into opposite directions within alternating layers of linker molecules. Note that, despite the arbitrariness of the initial structures, the generality of the results by means of the above described NNP‐based simulated annealing procedure is not affected.

The overall simulated annealing protocol is schematically depicted in Figure 2. In all instances, energy minimizations were carried out using the limited‐memory Broyden–Fletcher–Goldfarb–Shanno (L‐BFGS) algorithm [39] in conjunction with isotropic unit cell optimization, applying a force convergence criterion of 0.1 eV/Å. Firstly, the initial structures were optimized prior to starting the annealing procedure at a temperature of 5000 K, before cooling down to 1 K with the temperature being decreased by a factor of 0.995 in each MC step attempt, resulting in 1700 annealing steps. Secondly, a number of successive cooling cycles was executed, each time starting from the lowest‐energy structure found during all previous cycles. The respective structures were cooled down from 200 K to 1 K within each cycle, with a temperature decrease factor of 0.99 in each MC step attempt (approximately 1060 annealing steps). The total number of cycles was chosen by taking both energetic and structural convergence into consideration (*vide infra*).

The linker orientations were observed by means of the approach outlined above involving the 2‐bit binary sequences for each linker unit (see Figure 3). Global structural changes in the MOF systems were tracked and quantified via RMSD calculations [35] taking into account only the atoms comprising the inorganic nodes (excluding hydrogen atoms of the ${\text{Zr}}_{6}{\text{O}}_{4}{\text{(OH)}}_{4}^{12+}$ clusters in case of UiO‐66(Zr)‐NH_2_). For these RMSD calculations, the inorganic node atoms were translated as a whole towards their collective center of mass within each frame of the simulated annealing output (i.e., within each accepted MC step), most notably without performing rotations as it is reasonable for extensive crystalline solids such as MOFs, before calculating the RMSD on an atom‐by‐atom basis.

Finally, MD simulations were set up at ambient conditions for both the starting structures and lowest‐energy structures obtained from the simulated annealing procedures for MOF‐5‐OH, SNU‐70, and UiO‐66(Zr)‐NH_2_. The objectives of these simulations were to calculate both ensemble‐averaged PXRD patterns [12] as well as RDFs for multiple element pairs, that is M–C, M–O, and M–M, where either M = Zn (MOF‐5‐OH, SNU‐70) or Zr (UiO‐66(Zr)‐NH_2_). All simulations were performed utilizing the PQ software package [40] in conjunction with the NNP MACE‐MP‐0a (model size medium) for energy and force calculations, once again employing the DFT‐D3(BJ) dispersion correction scheme. The MD simulations were carried out for 100 ps (velocity Verlet integrator [41], time step 0.5 fs) within the *NPT* ensemble at *T* = 298.15 K (Bussi–Donadio–Parrinello velocity rescaling thermostat [42], relaxation time 0.1 ps) and *P* = 1.013 bar (Berendsen manostat [43], relaxation time 5.0 ps) after equilibrating for 10 ps under *NVT* and *NPT* conditions, respectively. The targeted ensemble‐averaged PXRD patterns were obtained by averaging multiple individual PXRD patterns (Cu Kα radiation; λ = 1.5406 Å) taken from single frames of the respective MD trajectories [12] (2000 equispaced frames from a 100 ps *NPT* MD simulation each). For each individual PXRD pattern calculation, a crystallographic information file (CIF) was created and forwarded to the RIETAN‐FP suite [44] provided by VESTA [45] to determine reflections. The latter were Gaussian‐broadened by means of a weighted kernel density estimation (KDE) [46] with a broadness factor of σ =0.07^∘^, where σ corresponds to the standard deviation. Finally, the aforementioned RDF calculations were performed within the PQAnalysis software package [47], using a bin size of Δ*r* = 0.05 Å, with r being the interatomic distance.

## 4 Results and Discussion

**FIGURE 4:**
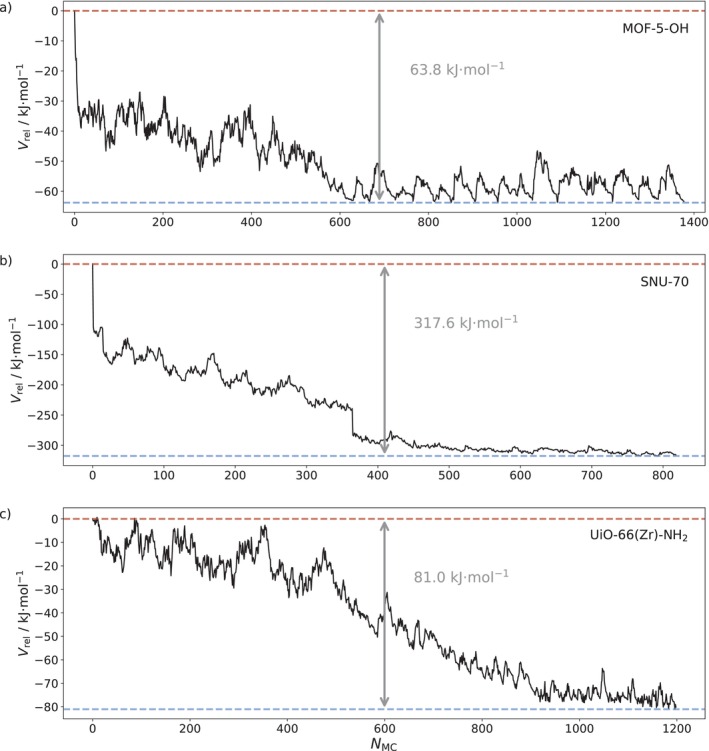
Evolution of the potential energy relative to the initial configuration over the course of the outlined NNP‐based simulated annealing procedure observed for (a) MOF‐5‐OH, (b) SNU‐70, and (c) UiO‐66(Zr)‐NH_2_.

The evolution of the potential energy over the NNP‐based simulated annealing procedure observed for the investigated MOF systems is shown in Figure 4. The energy decreased by 63.8, 317.6, and 81.0 kJ⋅mol^−1^ in case of MOF‐5‐OH, SNU‐70, and UiO‐66(Zr)‐NH_2_, respectively, before ultimately converging to a minimum.

Compared to the average thermal energy per degree of freedom available at ambient temperature (e.g., 298.15 K), that is RT≈2.5 $\text{kJ}\cdot {\text{mol}}^{-1}$ (where R and T are the ideal gas constant and temperature, respectively), the decrease in energy is substantial in all cases. Simulated annealing optimizations, as performed in this work, can in theory locate the global minimum, [26, 27] yet this is only possible when cooling from infinite temperature to absolute zero at minimal speed. Thus, as expected, the starting temperature and the cooling rates in both the first and all following thermocycles play a decisive role in determining the observed energy decrease in addition to the chosen random number seed. The utilized values summarized in Section 3 were chosen as a compromise between accuracy and computation time with the presented results corresponding to the runs with the lowest energies achieved for the considered MOF systems.

Due to the inherent randomness of the Metropolis algorithm [23], MC‐based studies are expected to yield different optimization trajectories when the initial random number generation settings vary. To assess the reproducibility of the simulated annealing framework, three additional annealing runs were performed for each investigated system using different random number seeds (see Supporting Information, Section S3). Despite the distinct paths taken through configurational space resulting from (i) different selections of linker molecules, (ii) different structural perturbations, and (iii) different random numbers entering the Metropolis criterion, the decrease in energy remained remarkably consistent. This indicates that the observed reduction in potential energy is driven by intrinsic structure–property relationships within the studied MOF systems.

The observed decrease in energy during the simulated annealing procedure should be discussed from a structural point of view, beginning with linker molecule orientations. The orientational distributions of the entirety of linker molecules in MOF‐5‐OH, SNU‐70, and UiO‐66(Zr)‐NH_2_ were tracked during the simulated annealing procedure via the 2‐bit binary representation outlined above (see Figure 3).

By doing so, the absolute number of rotated linker molecules after each accepted MC step was evaluated. The ordered starting structures of MOF‐5‐OH, SNU‐70, and UiO‐66(Zr)‐NH_2_ served as references, and both nodal and axial orientations were analyzed separately. In all cases, the number of rotated linker molecules increases rapidly, quickly approaching a value near 12, which corresponds to half of the total number of linker units in each case. Considering also the evolution of the potential energy shown in Figure 4, it can be concluded that the ordered initial structures are unfavorable, and the systems undergo rapid isomerization in the annealing process.

**FIGURE 5:**
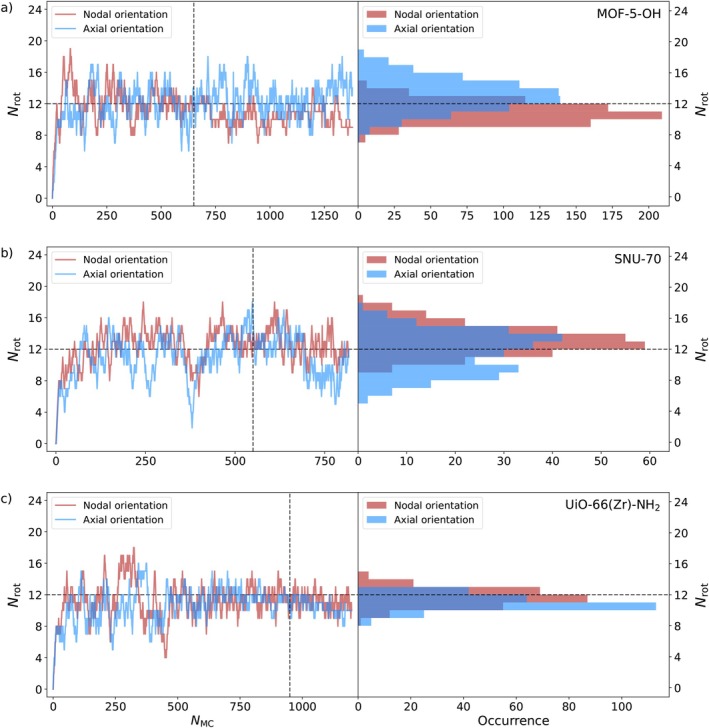
Evolution of the total number of rotated linker molecules $N_{\text{rot}}$ during the NNP‐based simulated annealing procedure (left) as well as accumulated histograms (right), starting the count at the MC step marked by the vertical dashed line, obtained for MOF‐5‐OH, (b) SNU‐70, and (c) UiO‐66(Zr)‐NH_2_. For visual orientation, horizontal dashed lines were added to indicate the 50/50 ratio in linker molecule orientations.

In addition to monitoring the linker orientation over the course of the MC‐based annealing, accumulated histograms for both the nodal and axial orientations are included in Figure 5, starting the count after the respective energies had approximately stabilized (see Figure 4), that is after ca. 650, 550, and 950 accepted MC steps in case of MOF‐5‐OH, SNU‐70, and UiO‐66(Zr)‐NH_2_, respectively. All three systems show Gaussian‐like profiles with a maximum around 12 rotated linker molecules (out of a total of 24) for both the nodal and axial orientations in the respective histograms. Even though the axial and nodal distributions are slightly skewed (axial orientations in SNU‐70) or offset from the exact value of 12 (nodal orientations in case of MOF‐5‐OH), taking into consideration multiple tested runs (see Supporting Information, Section S3) it was found that this deviation is within a certain margin of statistical variation as expected for a MC‐based optimizaton approach.

It is especially noteworthy that the energies of the MOF systems were continuing to decrease long after the numbers of rotated linker molecules had already stabilized (compare Figures 4 and 5). Crucially, the relative orientations of the linker molecules towards one another were still changing and thereby leading to lower energy values while, at the same time, leaving the total number of rotated linker molecules approximately constant. This implies that within each system, the lowest‐energy configurations arise due to cooperative effects associated with the linker molecules rather than purely stochastic orientations of individual linkers. In case of UiO‐66(Zr)‐NH_2_, this can be visually confirmed by the fact that the amino groups of the BDC‐NH_2_ linker molecules have the tendency to point towards each other, which could be viewed as (partial) hydrogen bonding [48, 49, 50] explaining the beneficial energetic effects.

**FIGURE 6:**
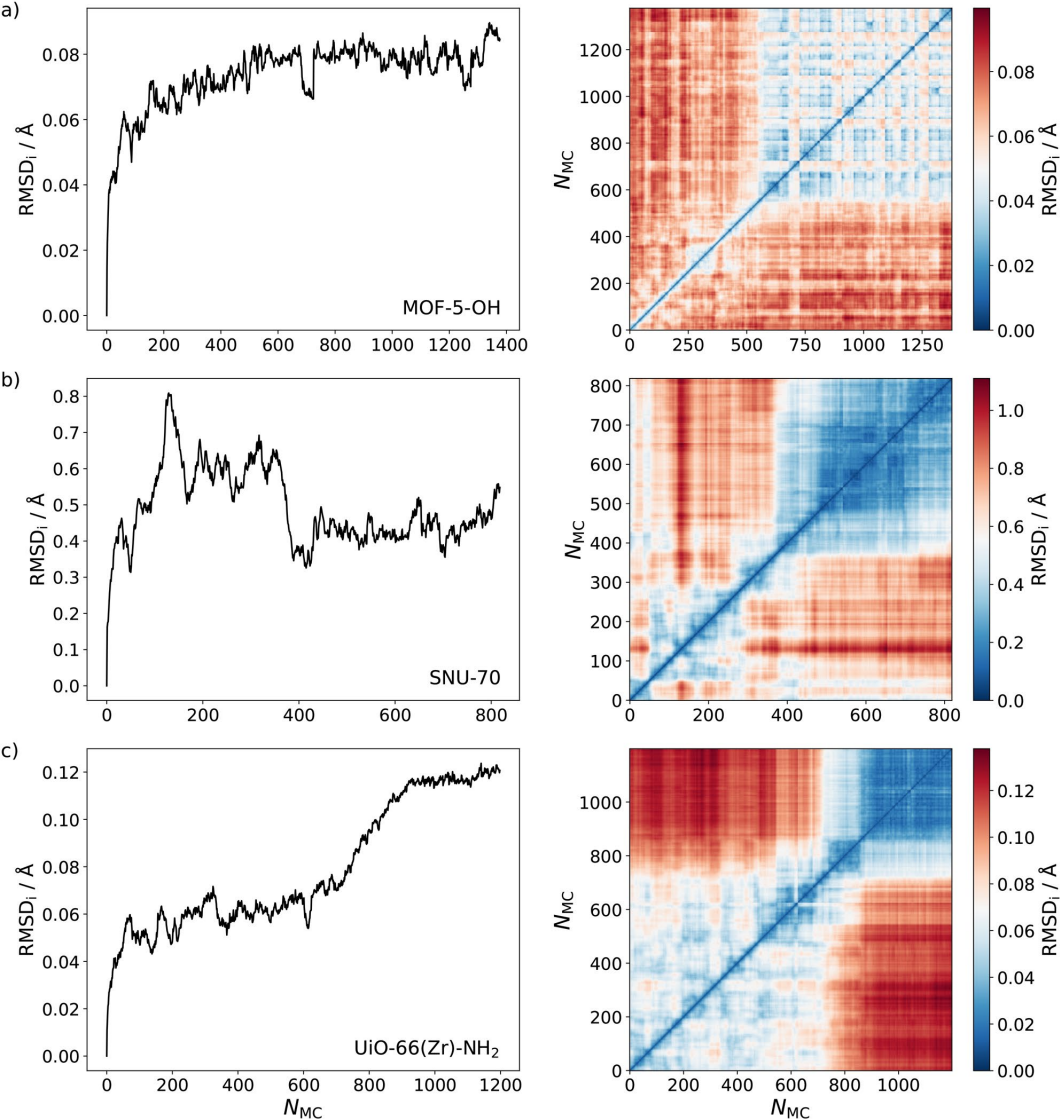
One‐ (left) and two‐dimensional (right) root‐mean‐square‐deviation plots, considering only the inorganic node atoms RMSD_i_ (excluding hydrogens) over the course of the NNP‐based simulated annealing procedure in case of (a) MOF‐5‐OH, (b) SNU‐70, and (c) UiO‐66(Zr)‐NH_2_.

The mentioned cooperative effects within the entirety of linker molecules furthermore cause small but notable changes in the global structure in all three MOF systems, which were identified and studied via RMSD calculations based solely on the atoms of the inorganic nodes (excluding hydrogens in case of the ${\text{Zr}}_{6}{\text{O}}_{4}{\text{(OH)}}_{4}^{12+}$ cluster of UiO‐66(Zr)‐NH_2_). For each system a one‐dimensional RMSD calculation relative to the initial structure along with full 2D RMSD analyses were carried out (see Figure 6), as described above.

It can be seen from both the one‐ and two‐dimensional RMSD representations that the structural resemblance with respect to the initial configuration decreases rapidly as the simulated annealing procedure progresses, indicating structural changes in the process. These structural changes perfectly align with the decrease in potential energy discussed above. In addition, having performed multiple thermocycles causes the appearance of pronounced patterns in the 2D RMSDs (e.g., along the main diagonal) in the sense that many structures from one and the same cycle are structurally similar to each other, as indicated by the low RMSD values. Lastly, the close similarity of a large portion of the latter structures among each other as visible from the off‐diagonal region in the 2D RMSDs indicates structural convergence of the MOF systems at the final stages of the simulated annealing procedure. This finding illustrates the benefit of carrying out a series of subsequent thermocycles in achieving structural convergence.

Although the observed RMSD values are comparably small, they show systematic trends in case of all three investigated MOF systems. It is important to remark that the RMSD calculations based solely on inorganic nodes have proven to be a highly sensitive probe for monitoring global structural changes arising from linker molecule manipulation.

Overall, MOF‐5‐OH and UiO‐66(Zr)‐NH_2_ show RMSD values of similar magnitude, while those of SNU‐70 are higher by approximately a factor of 8. This can be attributed to the different nature of the CVB linker units (see Figure 1c) that—in addition to the aromatic moiety—contain a short aliphatic chain. The reduced symmetry relative to BDC‐OH and BDC‐NH_2_ promotes more extensive structural relaxation of the inorganic nodes.

As another global structural feature, the evolution of the lattice parameters was monitored over the course of the simulated annealing procedures for all three investigated MOFs (see Supporting Information, Figure S2). The trends in the lattice parameters coincide with the observed inorganic node‐based RMSD values and stabilize towards the end of the runs. While the overall change of the lattice parameter is minor, it nevertheless provides another indication that variations in the linker orientation have a direct influence on the physicochemical properties of the systems.

Further RMSD calculations were performed between the ten lowest‐energy MOF structures identified for each system, this time considering both the inorganic building units and the linker molecules excluding hydrogen atoms (see Supporting Information, Figure S3). It can be seen that over the course of the simulated annealing procedure, identical and/or highly similar configurations, as far as linker orientations are concerned, can be identified. The latter underlines that an annealing strategy based on just a single thermocycle might not be sufficient to achieve a fully converged optimization.

All discussed energetic and structural properties clearly point towards the influence the instantaneous orientation of the linker molecules have on the properties of the systems. However, in addition to the presentation of the data obtained from the simulated annealing procedure, it was important to investigate whether ensemble‐averaged measurements are sensitive to distinguish between the initial (i.e., ordered) and optimized MOF systems. To address this question, NNP‐based MD simulations at standard conditions (i.e., 298.15 K, 1.013 bar) were carried out for all three investigated systems using both the ordered and optimized configurations as initial structures.

**FIGURE 7:**
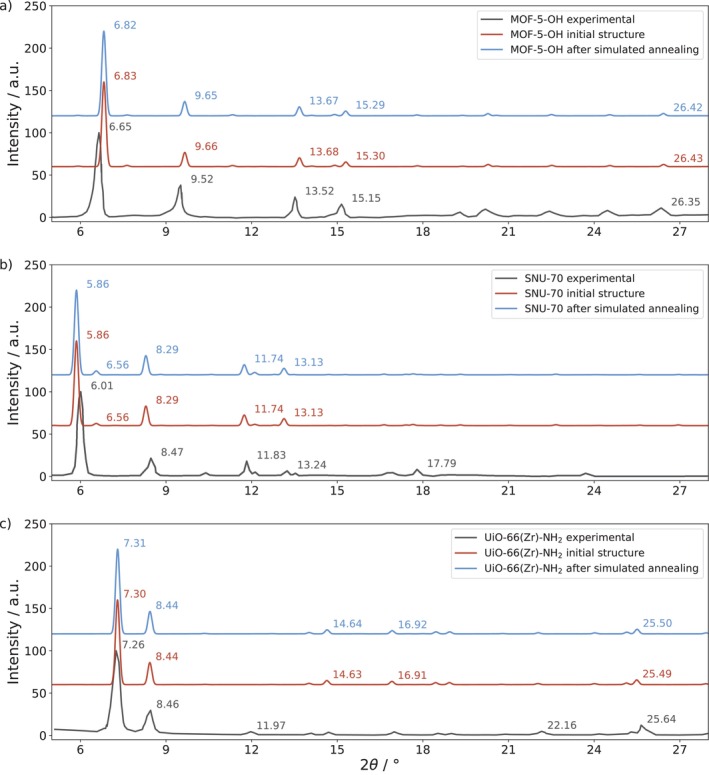
PXRD patterns (Cu Kα radiation) of (a) MOF‐5‐OH, (b) SNU‐70, and (c) UiO‐66(Zr)‐NH_2_. For each system, the computed ensemble‐averaged patterns (298.15 K, 1.013 bar) before and after performing the outlined NNP‐based simulated annealing procedure are compared to experimental data (digitized from original literature [17, 19, 51] using the Engauge Digitizer [52]). The positions of the five most intense reflections (i.e., 2θ values in degrees) are given for each pattern.

The ensemble‐averaged PXRD patterns determined for 2000 equispaced configurations per system are shown in Figure 7 and compared to experimental reference data [17, 19, 51]. The fact that the computed PXRD patterns for all three MOF systems are in excellent agreement with the previously reported experimental data, with very small noticeable deviations in case of MOF‐5‐OH and SNU‐70, hints at a sufficiently accurate structural description of the MOF systems via the employed NNP. In case of MOF‐5‐OH, the comparison to the PXRD data of the variant containing a molar fraction of 0.7 BDC‐OH has been considered, since the solvothermal synthesis employed by Kubo et al. could not yield a fully substituted MOF‐5‐OH system. This is of no practical concern however, as the authors have shown that the PXRD patterns obtained for different MOF‐5‐OH systems are effectively insensitive to the molar fraction of BDC‐OH (see Figure 1 in Reference [19]).

Comparison of the PXRD patterns obtained before and after simulated annealing shows that this analysis is not sufficiently sensitive to detect structural changes occurring at the microscopic level. This is to be expected since the overall symmetry of the MOF systems effectively remains unchanged upon re‐orientation of the linker molecules. Additionally, the collective structural modifications in the inorganic nodes, observed in the RMSD results, are too subtle to be resolved by PXRD, as they are minor compared to thermal vibrations of the individual atoms at standard conditions.

**FIGURE 8:**
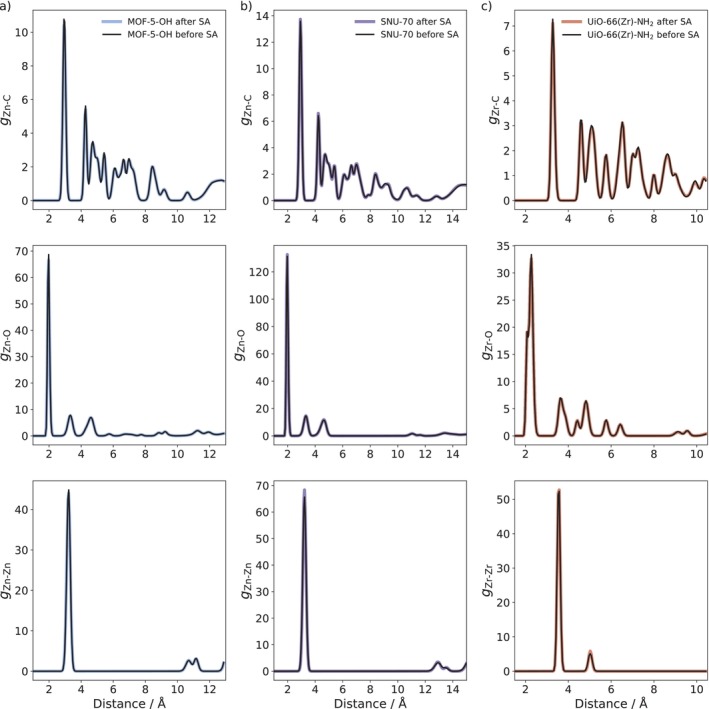
RDFs of different element pairs for (a) MOF‐5‐OH, (b) SNU‐70, and (c) UiO‐66(Zr)‐NH_2_. For each system, the pair distributions were computed from the MD simulation trajectories performed at ambient conditions (298.15 K, 1.013 bar), both before and after the outlined NNP‐based simulated annealing procedure.

In addition to the analysis of the PXRD data, the RDFs of key element pairs are depicted in Figure 8, again comparing the MD results obtained for the initial linker configurations with those calculated using the optimized structures obtained from the simulated annealing procedure. Also in this case, the RDFs before and after the simulated annealing procedures are not sensitive to the observed structural changes. Thus, also the local (i.e., short‐range) order is not significantly disturbed by linker molecule re‐orientations.

## 5 Conclusion

In this work, an NNP‐based simulated annealing approach was applied to three MOF systems, namely MOF‐5‐OH, SNU‐70, and UiO‐66(Zr)‐NH_2_, each containing low‐symmetry linker molecules causing ambiguities as far as linker orientation is concerned. Thus, the ultimate goal of this work was to approach the global minimum MOF structure associated with linker molecule‐induced orientational isomerism. By employing the state‐of‐the‐art NNP MACE‐MP‐0a (model size medium) for energy and force calculations, the developed simulated annealing approach was efficiently applied to identify structures close to—or ideally at—the global minimum. This assessment is supported by both energetic and structural convergence criteria, namely the observed potential energy decrease and different RMSD analyses. Based on the obtained results it can be concluded that cooperative effects arising from linker molecule orientation have a strong influence on the potential energy of the MOF system and minor effects on its structure, as shown by inorganic node‐based RMSD calculations, theoretically calculated PXRD patterns as well as the RDF data. The accuracy and suitability of the utilized NNP MACE‐MP‐0a (model size medium) is underlined by the excellent agreement of the computed MD‐based PXRD patterns with previously recorded experimental data.

The relevance of studying orientational isomerism in a computational fashion as done in this work is especially highlighted by the fact that while energetically the discussed linker re‐orientations are not to be neglected, typical experimental analysis methods such as PXRD or RDF evaluations are not able to distinguish between different isomeric forms due to the small overall structural effects. On the other hand, from a computational standpoint, the determination of RMSD values based solely on the atomic positions associated to the inorganic nodes proved as a sensible tool to monitor the structural rearrangements.

As a potential out‐of‐the‐box extension of the presented study, the effects of incorporating guest molecules on the linker orientations (e.g., hydrogen gas within MOF‐5‐OH [19] and SNU‐70 [53] or various types of drug molecules embedded in UiO‐66(Zr)‐NH_2_ [54, 55, 56]) could be investigated using the outlined simulated annealing procedure. These investigations would reflect—at the atomic level—a synthetic strategy with guest molecules being transferred into the MOF pores (i.e., host material) during MOF assembly [57] (instead of thereafter). Preliminary testing has revealed an apparent influence of the guest molecule's size and/or polarity on the achievable minimum energy of the pristine MOF systems, indicating that large guest molecules hinder the development of a collectively beneficial linker orientation by strong local influence leading to enhanced guest@MOF binding.

Furthermore, extending the applied simulated annealing framework to more complex MOF compounds is straightforward as well. Typical exemplary MOFs containing non‐planar linker molecules such as 2‐chloro‐ and 2‐nitrobenzene‐1,4‐dicarboxylate (BDC‐Cl and BDC‐NO_2_), feature aromatic rings and carboxylate groups that form dihedral angles different from 0° or 180°. Since this dihedral deviation can occur above or below the reference plane defined by the carboxylate groups, an additional MC step involving the permutation of dihedral angles must be included. This step requires more informaton from the user, however, as the rotatable subunits and approximate dihedral angles must be specified upon input.

Another extension involves investigating MOF compounds containing only a partial molar fraction of asymmetric linkers. Beyond the orientational isomerism examined in this work, the possibility of swapping molecules with and without functional groups must also be considered. For example, in MOF‐5‐OH with a molar fraction of 0.5, the framework consists of 12 BDC‐OH and 12 unsubstituted BDC linker molecules. Due to the structural similarity of these linkers, the placement of the carboxylate groups can serve as a marker when swapping BDC‐OH and BDC within the structure.

More complex linkers that connect to three or more inorganic building units such as asymmetrically substituted benzene tricarboxylate (BTC) may present a greater challenge. In these cases, the permutations of linker orientations often cannot be represented simply by mirroring or rotation. Instead, they involve higher‐order symmetry operations such as rotational reflections.

Although the described expansions to also include more intricate MOF systems introduces increased complexity in linker management and often requires more detailed user input, the simulated annealing strategy presented in this work can be applied without modifying the core routines that execute the individual thermocycles. Potential extensions of the program are currently in development and will enable the investigation of a broad range of complex MOF systems already in the near future.

## Funding

This work was supported by the Austrian Science Fund (Grant No. P 35427).

## Supporting information




**Data S1.** Supporting Information.

## Data Availability

The developed simulated annealing algorithm, interfaced with the NNP MACE‐MP‐0a, is available via GitHub at https://github.com/HoferLab/SimulatedAnnealingLinkerMOF.
