# Supplementary material for: Tackling Orientational Isomerism in Metal–Organic Frameworks Comprising Low‐Symmetry Linker Molecules via Simulated Annealing Featuring a Neural Network Potential
Source: J Comput Chem. 2026 Apr 2;47(9):e70349. doi: 10.1002/jcc.70349 (PMC13045335; doi:10.1002/jcc.70349)
Supplement: Supplementary file 1 — Data S1. Supporting Information. [file JCC-47-0-s001.pdf]

Supporting Information: Tackling Orientational Isomerism in Metal–Organic Frameworks Comprising Low-Symmetry Linker Molecules via Simulated Annealing Featuring a Neural Network Potential

Benedikt E. Hörfarter,[†,‡] Stefan Seiwald,[†,‡] Clemens Hofstötter,[†] Armin Penz,[†] Josef M. Gallmetzer,[†] and Thomas S. Hofer[*,†]

†*Institute of General, Inorganic and Theoretical Chemistry, University of Innsbruck, Innrain 80–82, 6020 Innsbruck, Austria*

‡*B. E. Hörfarter and S. Seiwald contributed equally to this work.*

E-mail: T.Hofer@uibk.ac.at

Phone: +43-512-507-57111. Fax: +43-512-507-57199

S1 Experimental Structure of SNU-70

Figure S1 depicts the experimentally determined structure of the SNU-70 MOF compound. Due to orientational isomerism of the (E)-4-(2-carboxylatovinyl)benzoate (CVB) linker molecule, only a superposition of the four possible linker orientations can be resolved. This limitation poses a severe challenge when setting up the SNU-70 system in a computational study.

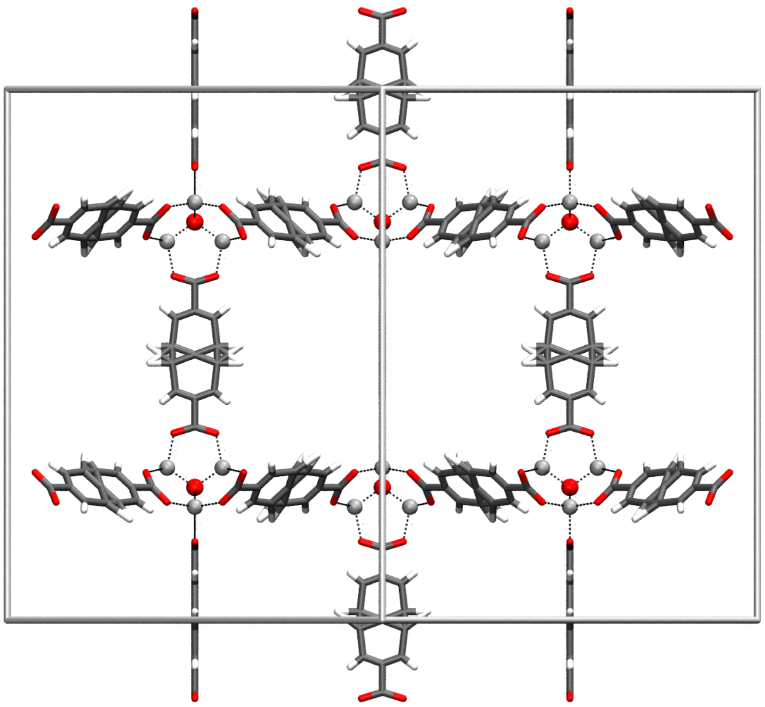

Figure S1: Experimentally determined structure of SNU-70 (CCDC-846935) viewed along the [110] direction.

S2 Additional Structural Description

Figures S2 and S3, respectively, show the evolution of the lattice parameters and additional root-mean-square-deviation (RMSD) data for MOF-5-OH, SNU-70 as well as UiO-66(Zr)-NH_2 during the Monte Carlo (MC)-based simulated annealing runs discussed in the main article.

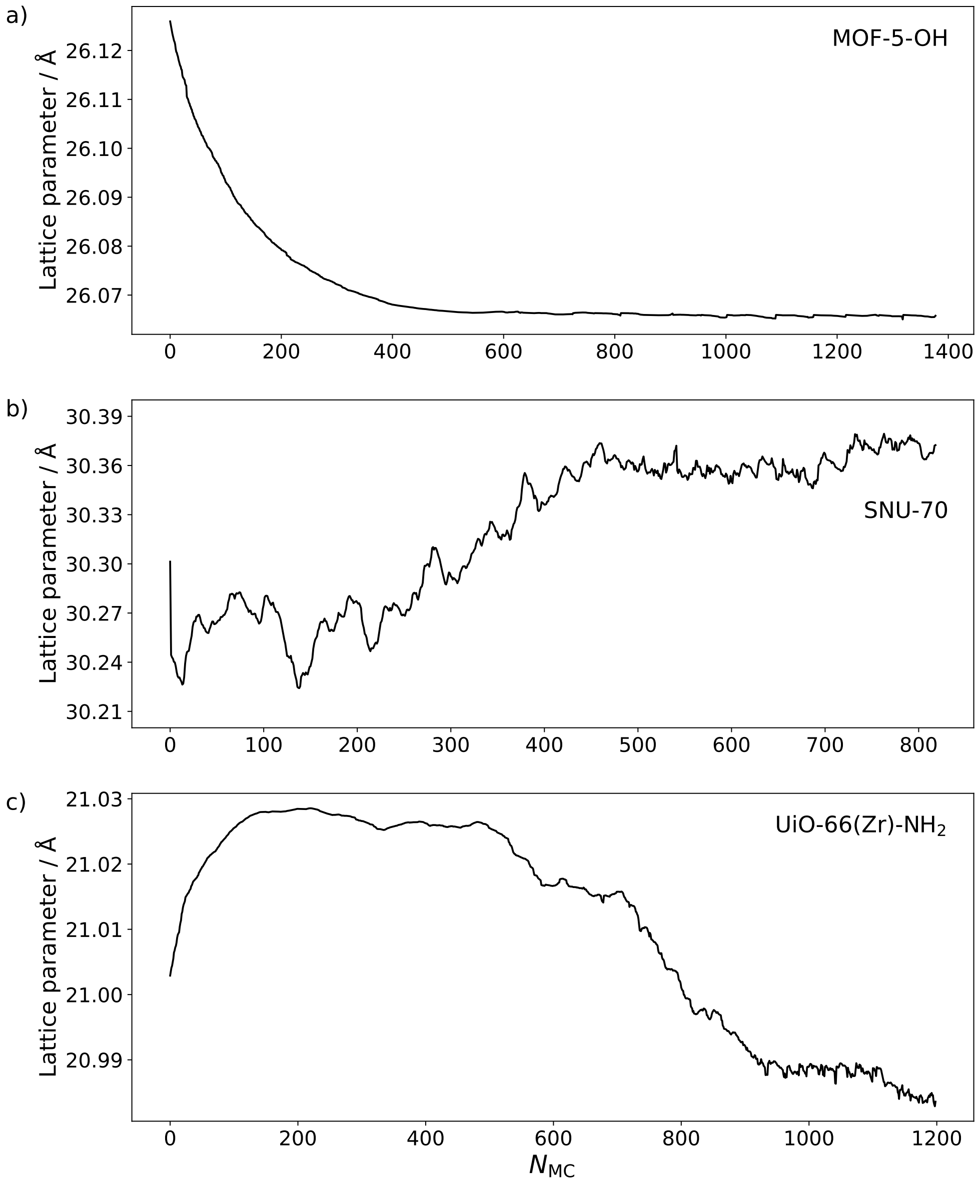

Figure S2: Evolution of the lattice parameters over the course of the MC-based simulated annealing procedures for a) MOF-5-OH, b) SNU-70 and c) UiO-66(Zr)-NH_2 as described in the main article.

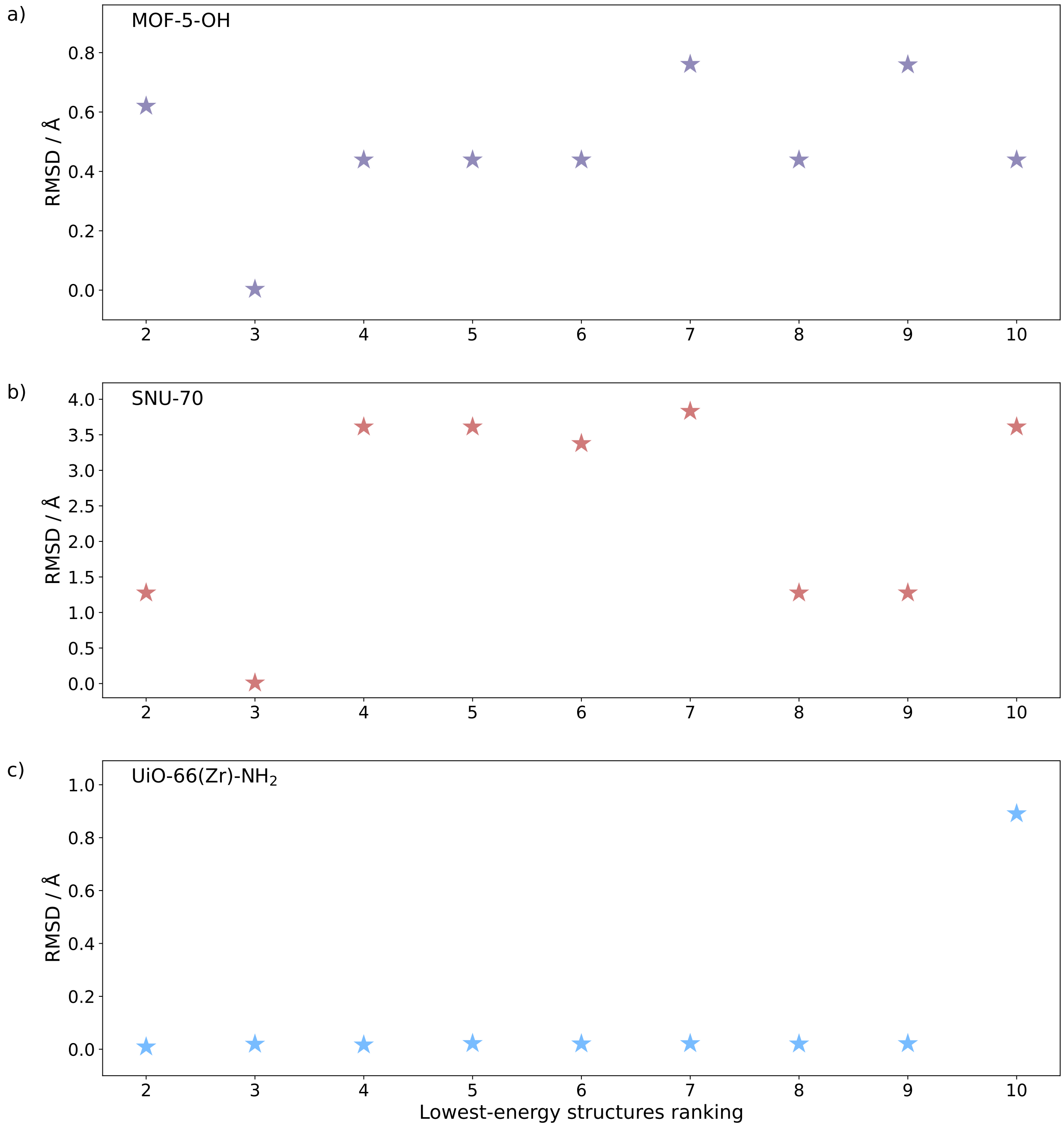

Figure S3: RMSD (excluding hydrogen atoms) of the lowest-energy structures (ranked second to tenth) found during the MC-based simulated annealing procedures for a) MOF-5-OH, b) SNU-70 and c) UiO-66(Zr)-NH_2 as described in the main artilce, taking the structure of lowest energy as reference.

S3 Results of Additional Simulated Annealing Runs

The results of three additionally performed simulated annealing runs for MOF-5-OH, SNU-70 and UiO-66(Zr)-NH_2 are shown in the following three Subsections S3.3–**??**, each time employing a different random seed with otherwise identical settings as described in the main text.

For each individual run, that is within each Subsection S3.3–**??**, the energy and linker orientation developments are depicted alongside the lattice parameter evolution as well as root mean square deviation (RMSD) calculations.

S3.1 Additional Simulated Annealing Runs of MOF-5-OH

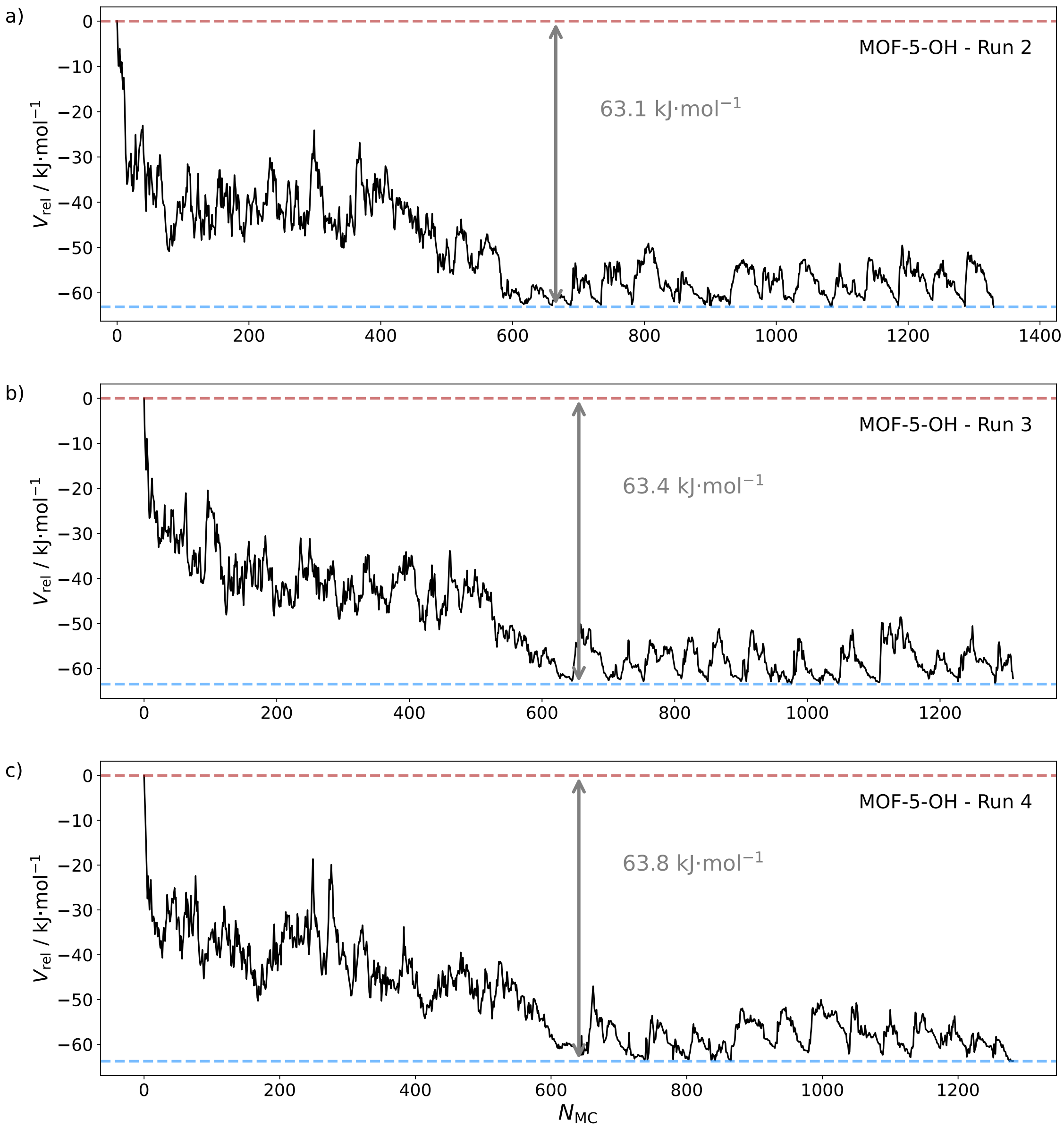

Figure S4: Evolution of the potential energy over the course of three different NNP-based simulated annealing runs for MOF-5-OH using the same initial structure with different random seed settings.

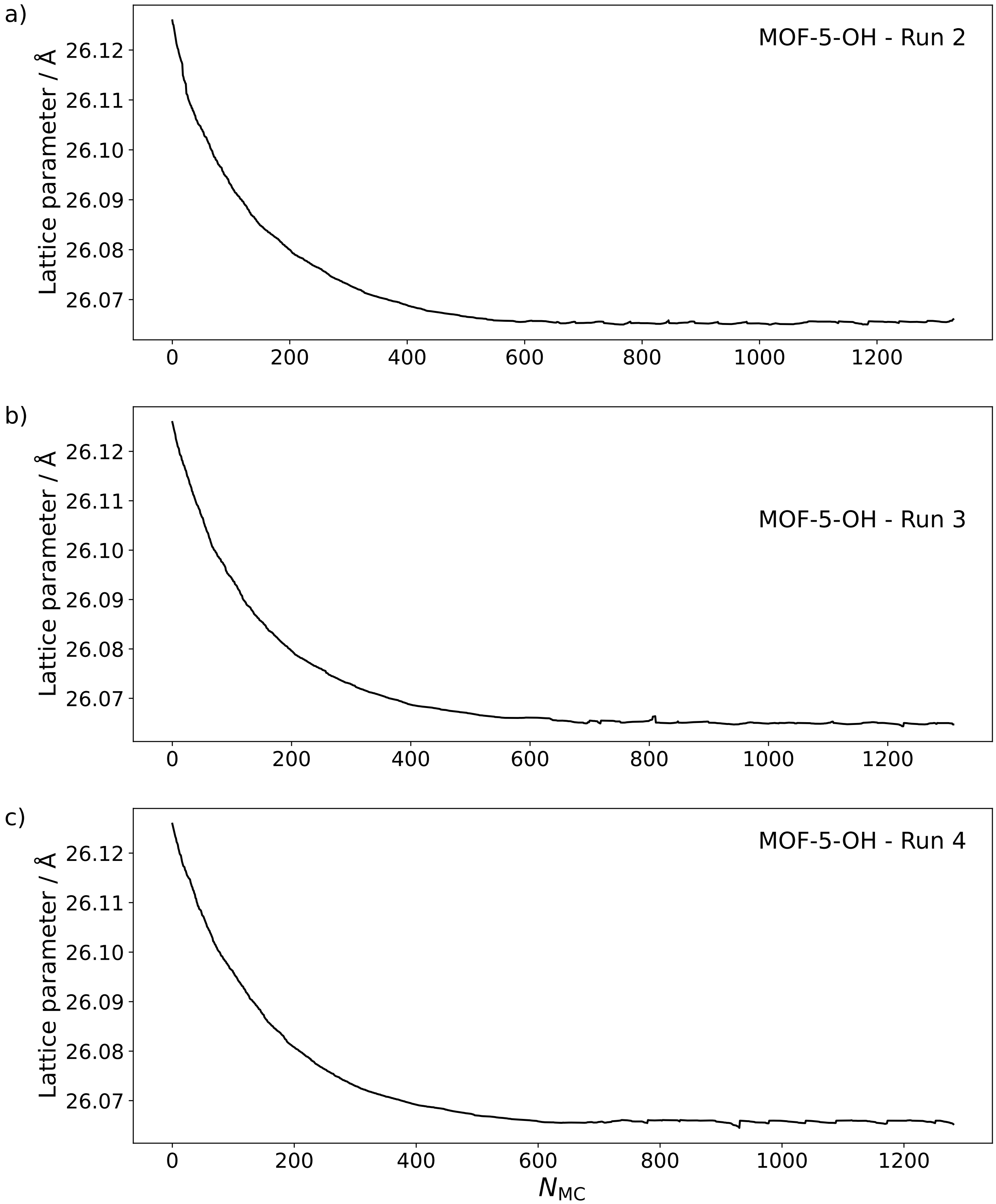

Figure S5: Evolution of the lattice parameter energy over the course of three different NNP-based simulated annealing runs for MOF-5-OH using the same initial structure with different random seed settings.

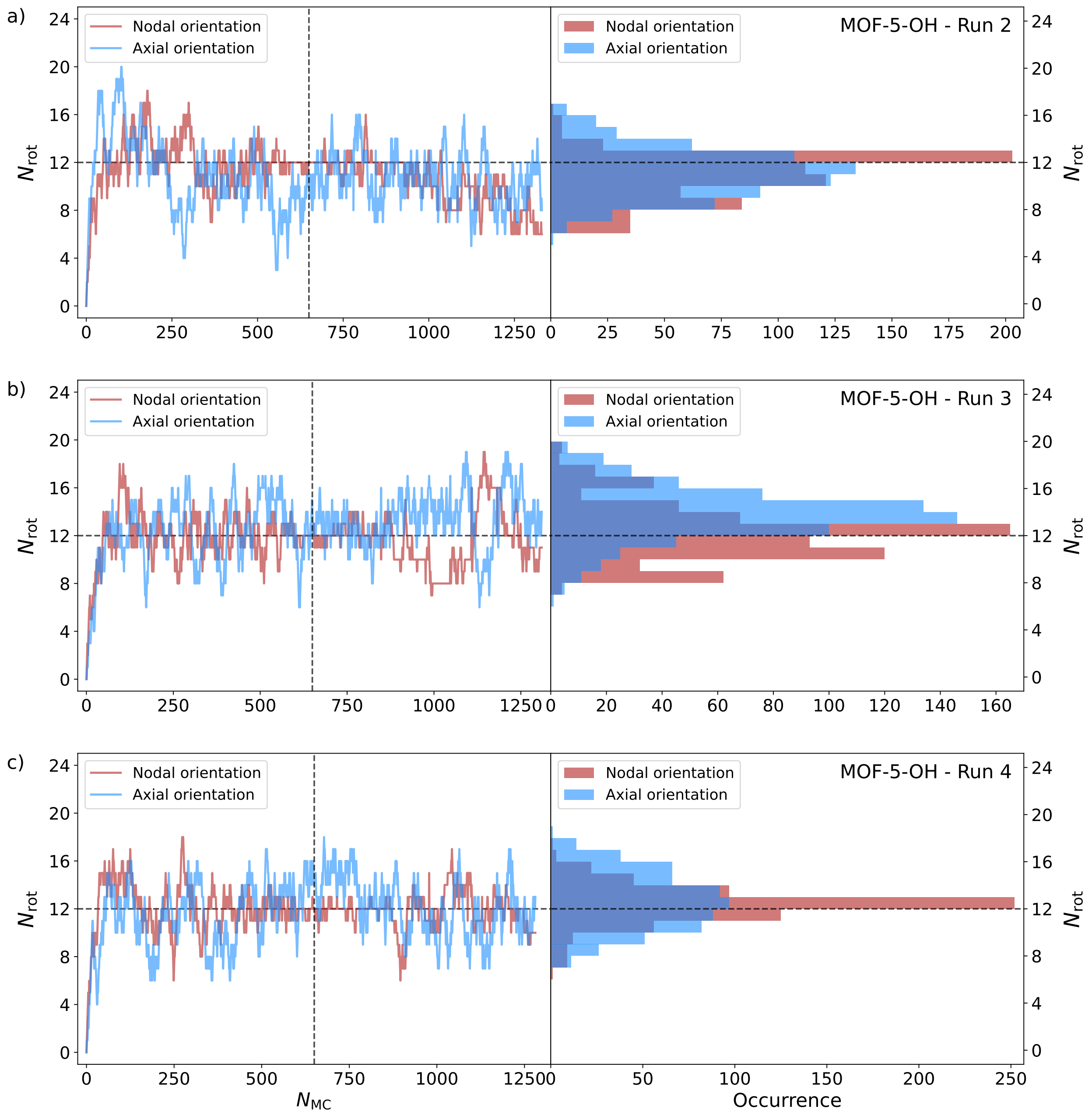

Figure S6: Evolution of the total number of rotated linker molecules during the NNP-based simulated annealing procedure (left) as well as accumulated histograms (right), starting the count at the MC step marked by the vertical dashed line for MOF-5-OH using the same initial structure with different random seed settings. For visual orientation, gray horizontal dashed lines were added which indicate a 50/50 ratio in linker molecule orientations.

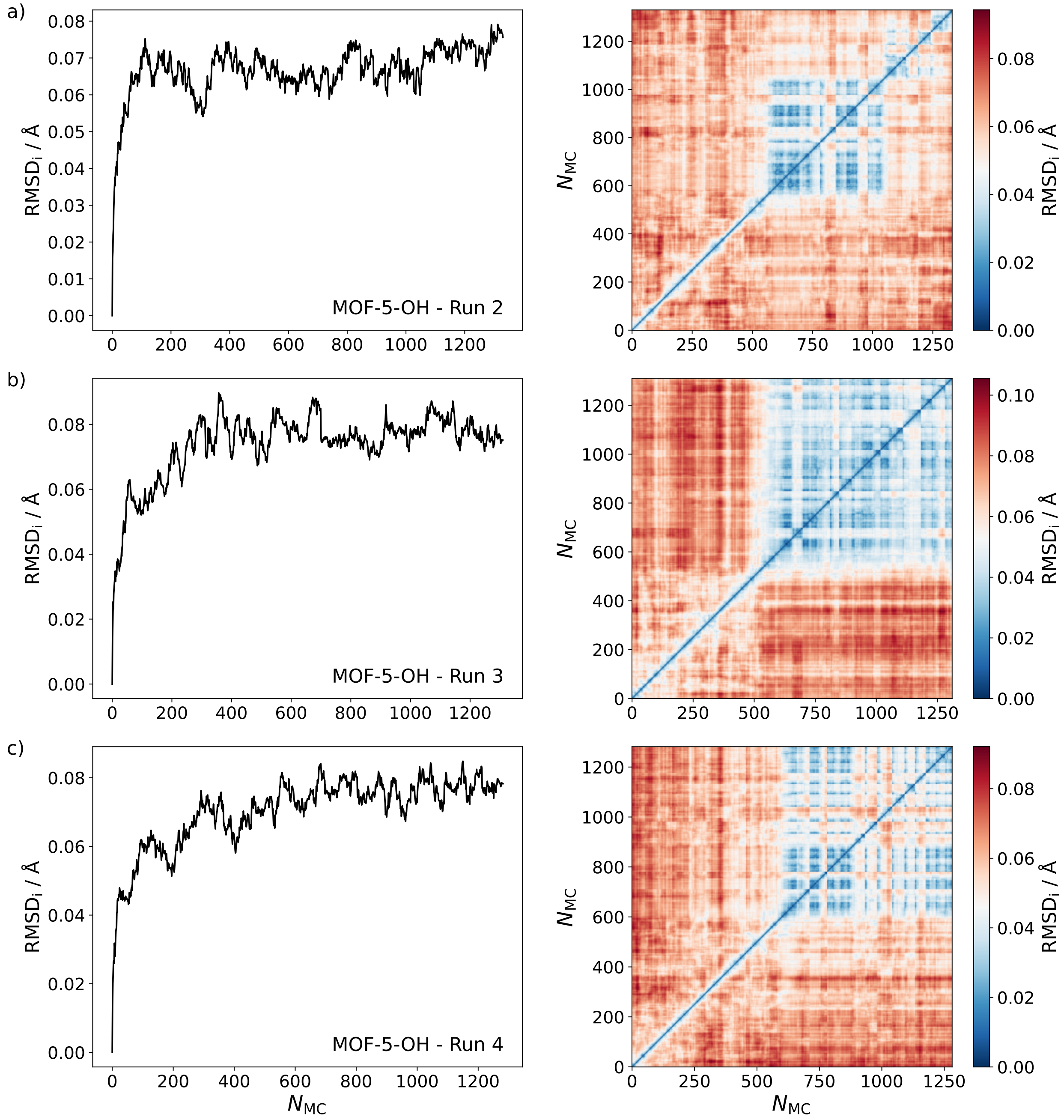

Figure S7: One- (left) and two-dimensional (right) root-mean-square-deviation plots, considering only the inorganic node atoms over the course of the NNP-based simulated annealing procedure for MOF-5-OH using the same initial structure with different random seed settings.

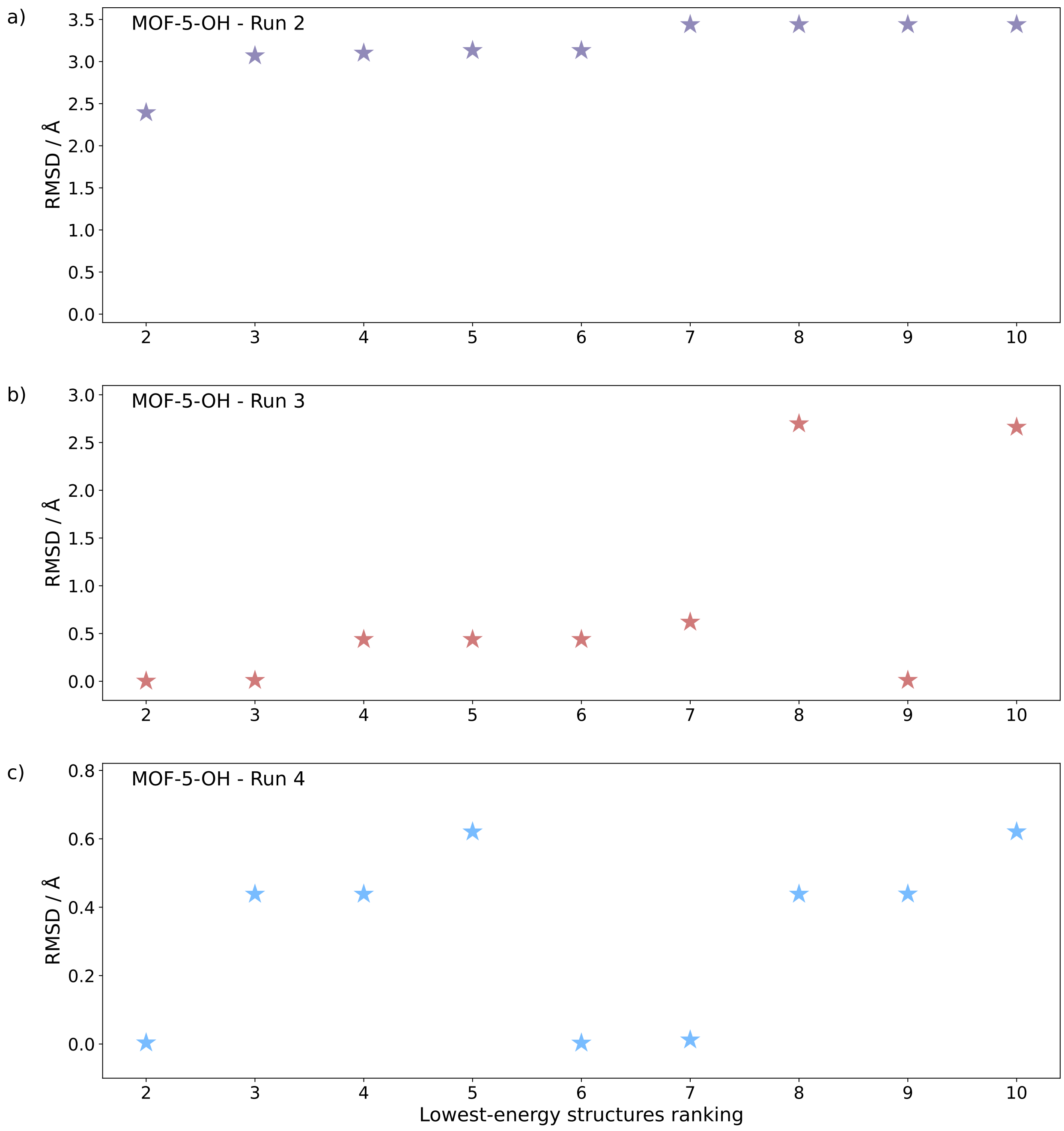

Figure S8: RMSD (excluding hydrogen atoms) of the lowest-energy structures (ranked second to tenth) found during the MC-based simulated annealing procedures for MOF-5-OH using the same initial structure with different random seed settings.

S3.2 Additional Simulated Annealing Runs of SNU-70

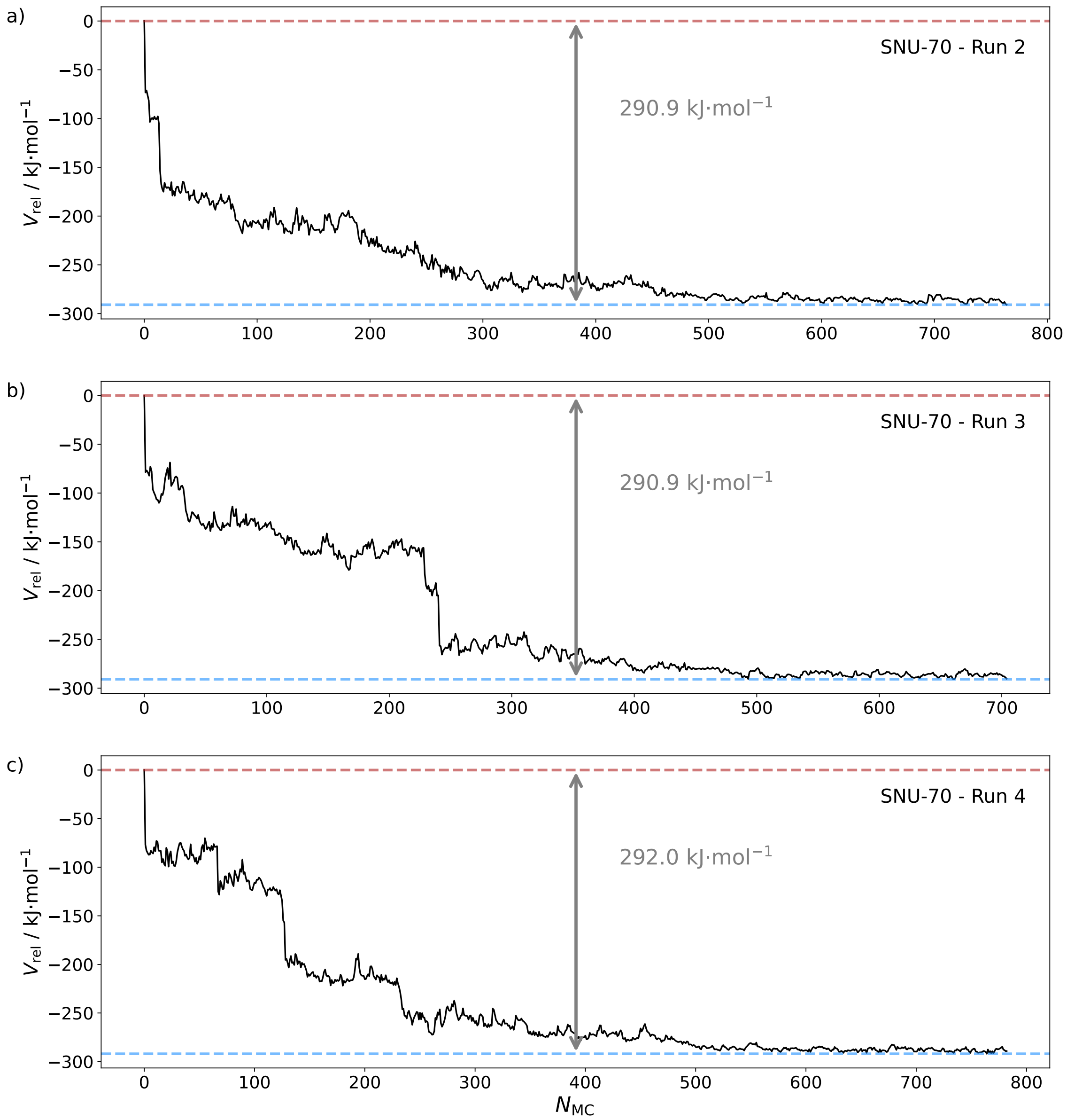

Figure S9: Evolution of the potential energy over the course of three different NNP-based simulated annealing runs for SNU-70 using the same initial structure with different random seed settings.

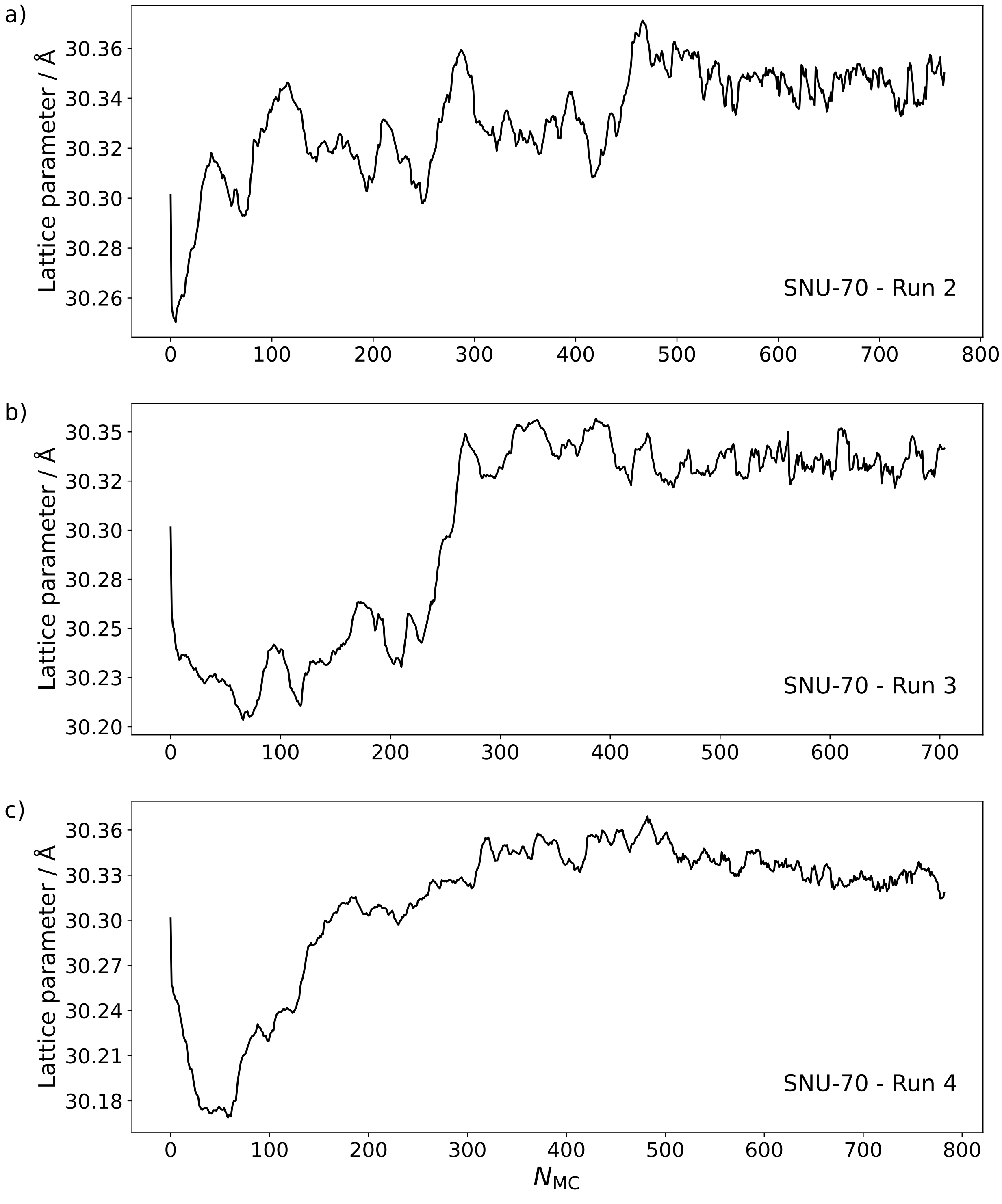

Figure S10: Evolution of the lattice parameter energy over the course of three different NNP-based simulated annealing runs for SNU-70 using the same initial structure with different random seed settings.

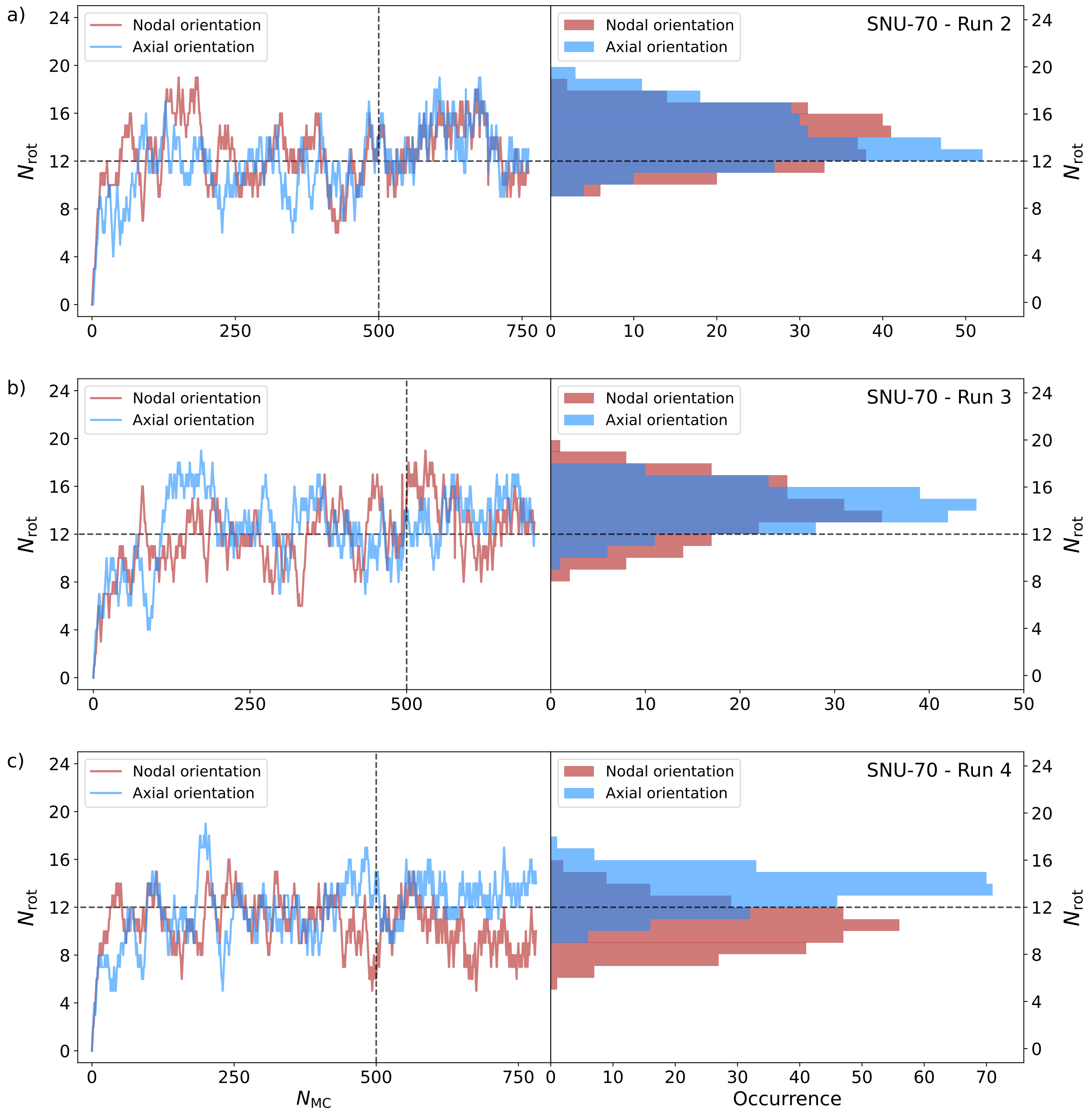

Figure S11: Evolution of the total number of rotated linker molecules during the NNP-based simulated annealing procedure (left) as well as accumulated histograms (right), starting the count at the MC step marked by the vertical dashed line for SNU-70 using the same initial structure with different random seed settings. For visual orientation, gray horizontal dashed lines were added which indicate a 50/50 ratio in linker molecule orientations.

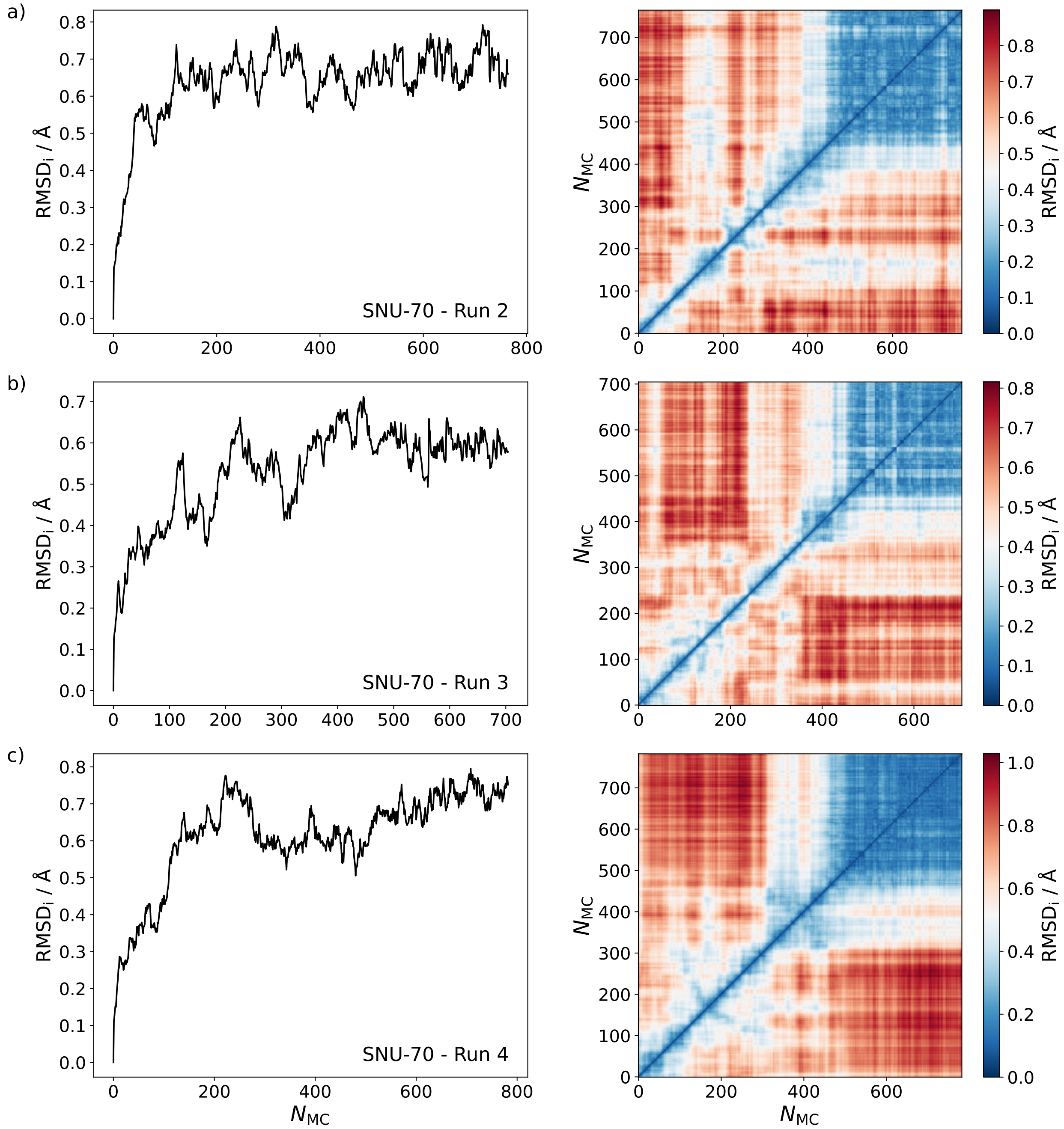

Figure S12: One- (left) and two-dimensional (right) root-mean-square-deviation plots, considering only the inorganic node atoms over the course of the NNP-based simulated annealing procedure for SNU-70 using the same initial structure with different random seed settings.

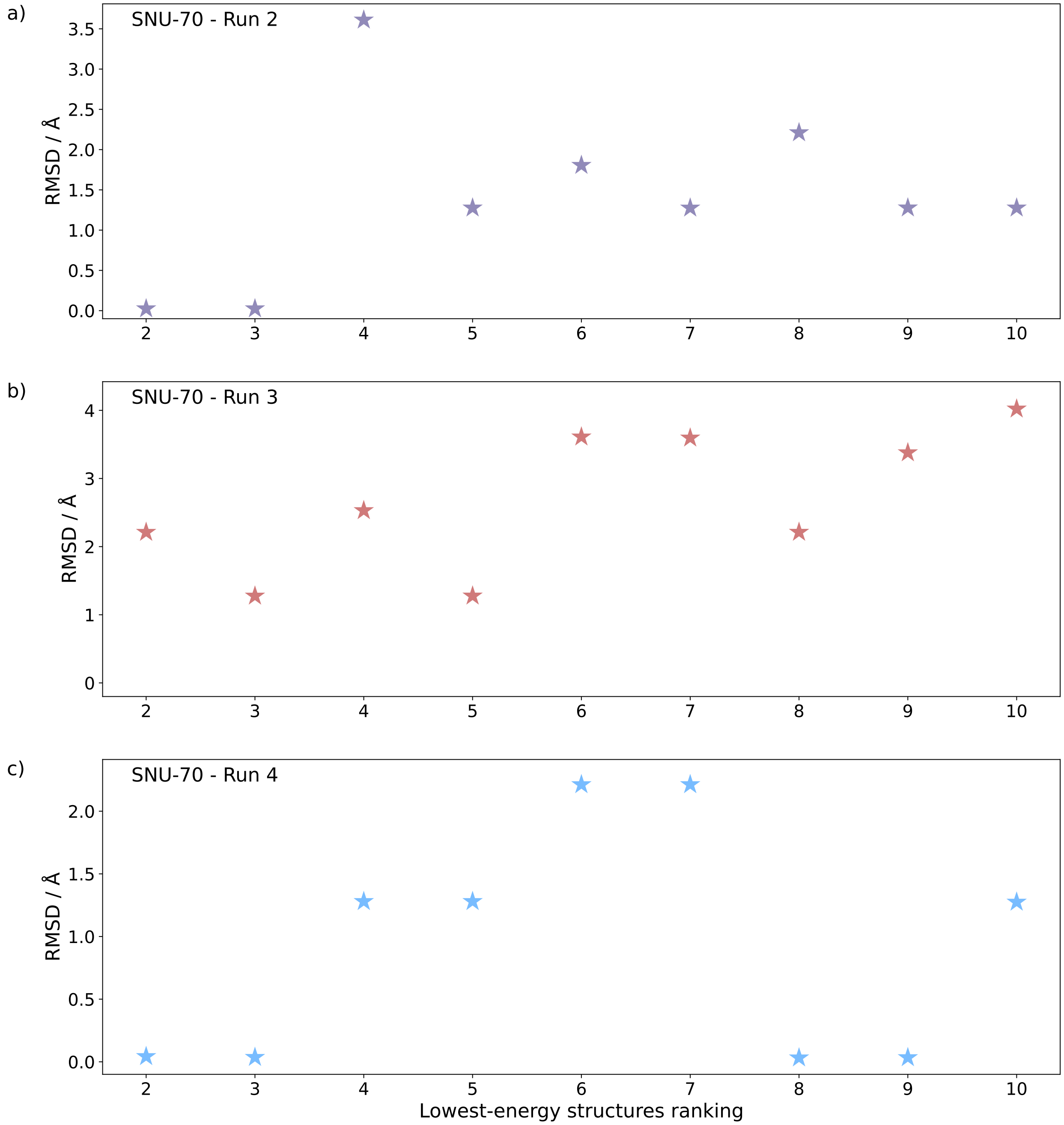

Figure S13: RMSD (excluding hydrogen atoms) of the lowest-energy structures (ranked second to tenth) found during the MC-based simulated annealing procedures for SNU-70 using the same initial structure with different random seed settings.

S3.3 Additional Simulated Annealing Runs of UiO-66(Zr)-NH_2

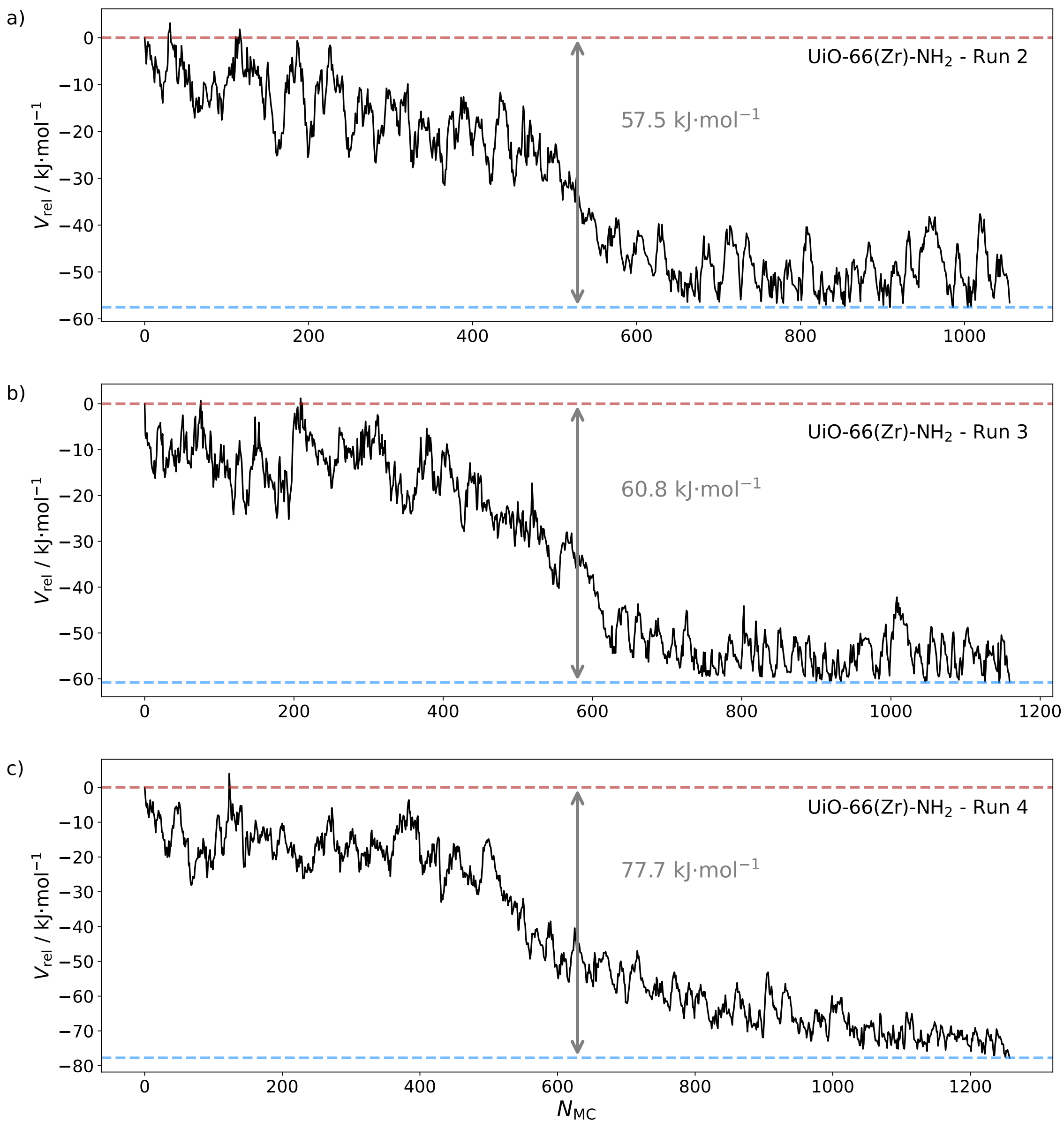

Figure S14: Evolution of the potential energy over the course of three different NNP-based simulated annealing runs for UiO-66(Zr)-NH_2 using the same initial structure with different random seed settings.

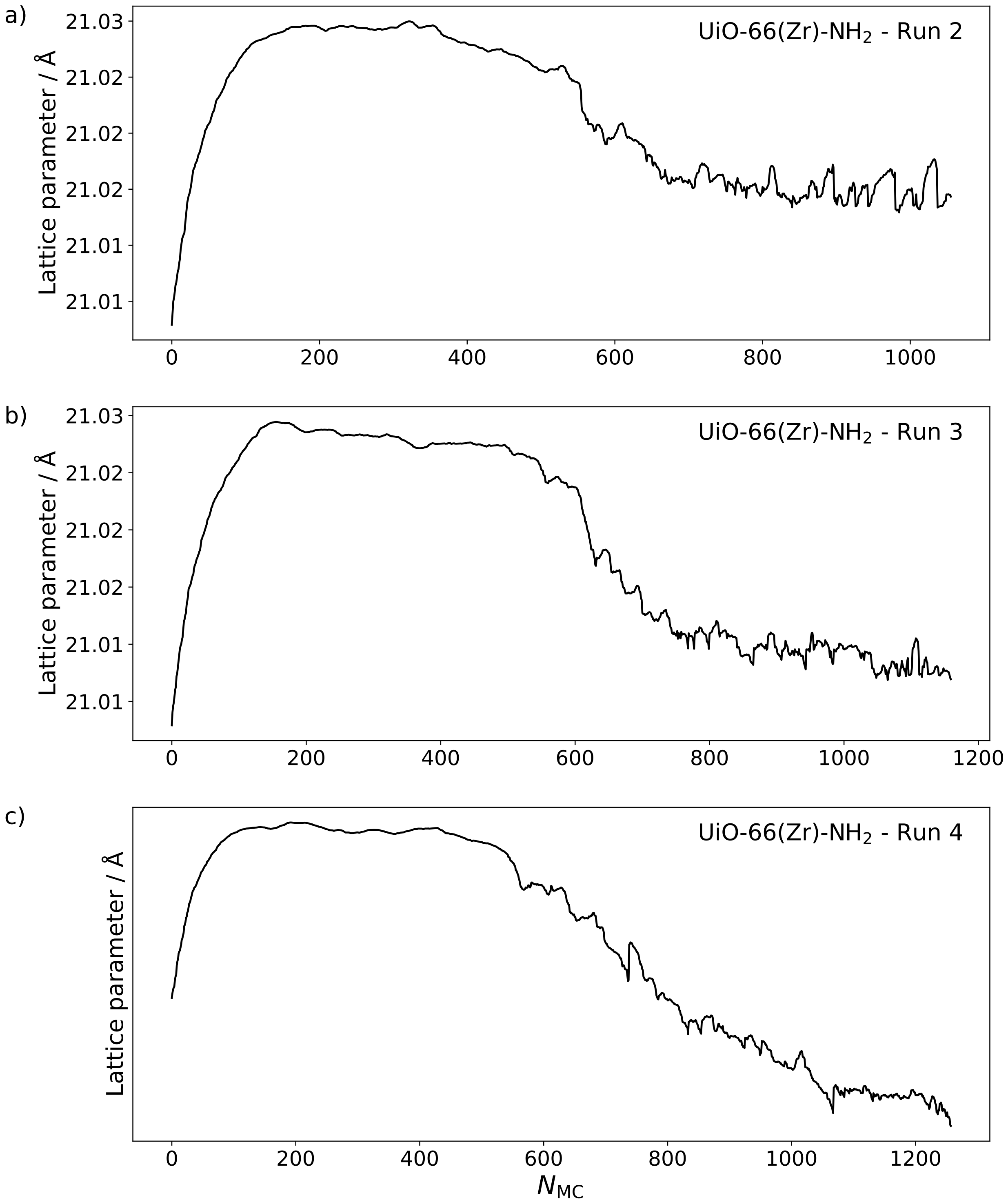

Figure S15: Evolution of the lattice parameter energy over the course of three different NNP-based simulated annealing runs for UiO-66(Zr)-NH_2 using the same initial structure with different random seed settings.

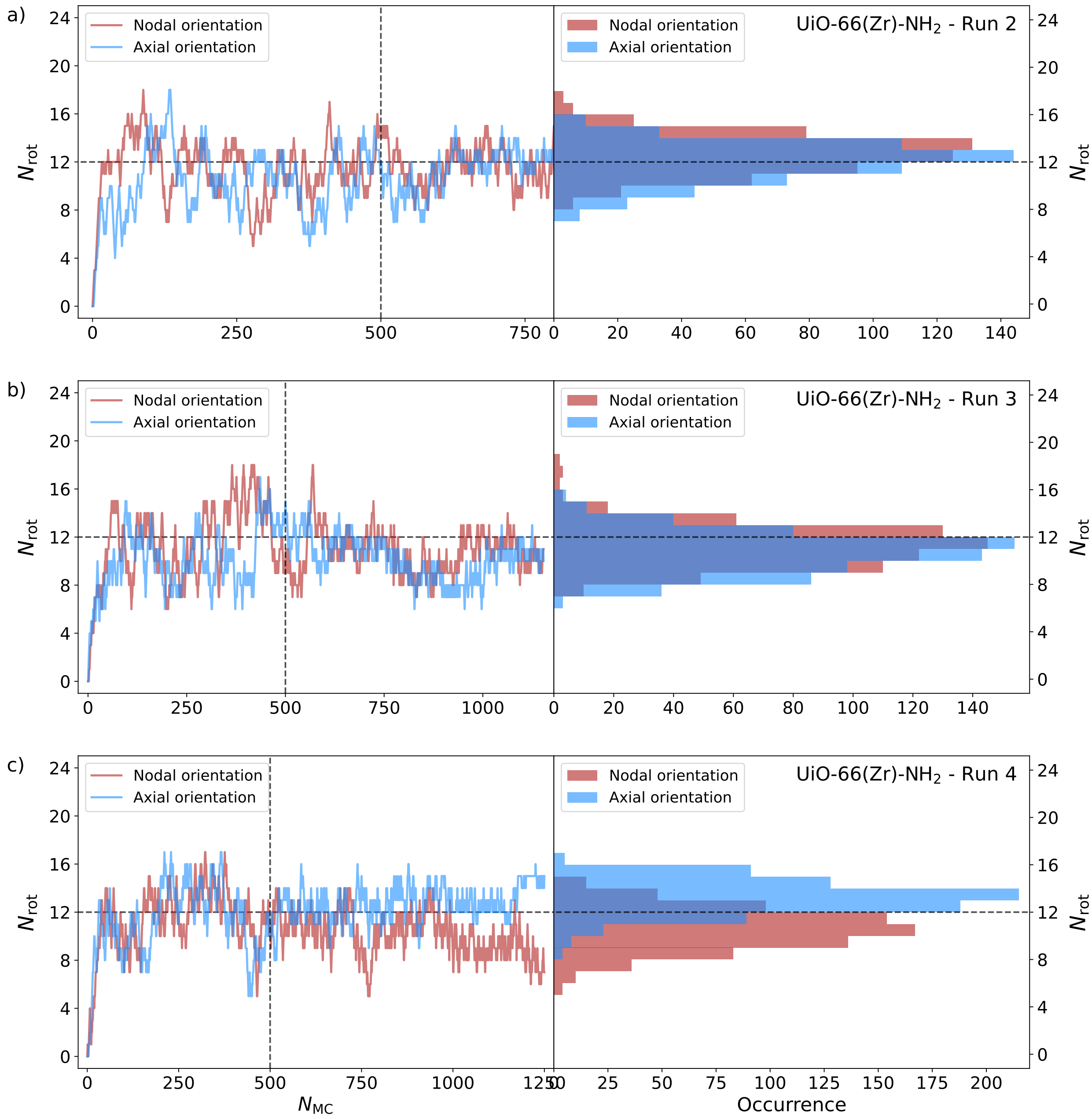

Figure S16: Evolution of the total number of rotated linker molecules during the NNP-based simulated annealing procedure (left) as well as accumulated histograms (right), starting the count at the MC step marked by the vertical dashed line for UiO-66(Zr)-NH_2 using the same initial structure with different random seed settings. For visual orientation, gray horizontal dashed lines were added which indicate a 50/50 ratio in linker molecule orientations.

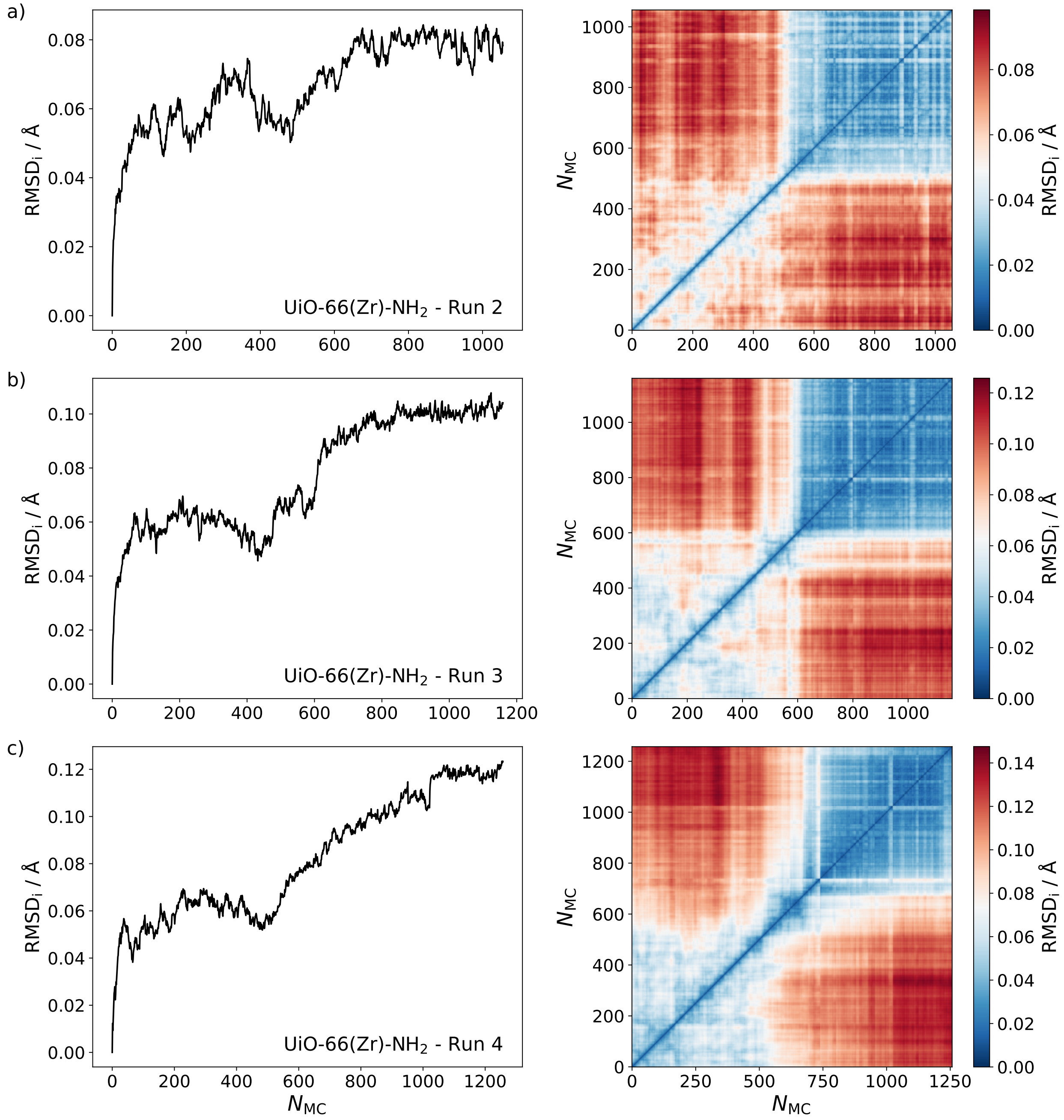

Figure S17: One- (left) and two-dimensional (right) root-mean-square-deviation plots, considering only the inorganic node atoms over the course of the NNP-based simulated annealing procedure for UiO-66(Zr)-NH_2 using the same initial structure with different random seed settings.

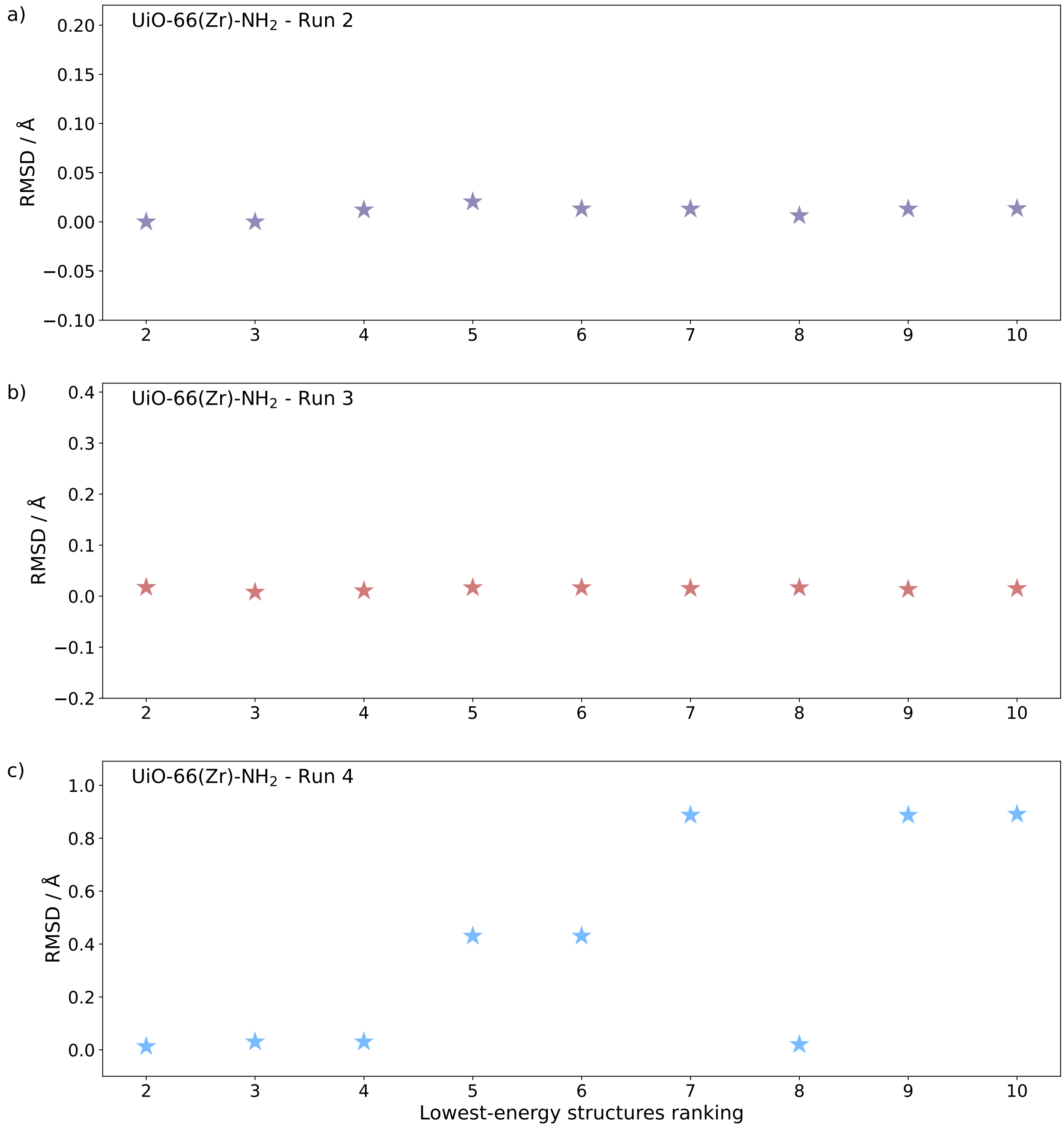

Figure S18: RMSD (excluding hydrogen atoms) of the lowest-energy structures (ranked second to tenth) found during the MC-based simulated annealing procedures for UiO-66(Zr)-NH_2 using the same initial structure with different random seed settings.

Author Information

B. E. Hörfarter and S. Seiwald contributed equally to this work.
